# Two mutually exclusive evolutionary scenarios for allexiviruses that overcome host RNA silencing and autophagy by regulating viral *CRP* expression

**DOI:** 10.1371/journal.ppat.1011457

**Published:** 2023-06-28

**Authors:** Hangil Kim, Shusuke Kawakubo, Haruna Takahashi, Chikara Masuta

**Affiliations:** Research Faculty of Agriculture, Hokkaido University, Kita-ku, Kita 9, Nishi 9, Sapporo, Japan; China Agricultural University, CHINA

## Abstract

The genus *Allexivirus* currently includes eight virus species that infect allium plants. Previously, we showed that there are two distinct groups of allexiviruses (deletion [D]-type and insertion [I]-type) based on the presence or absence of a 10- to 20-base insert (IS) between the coat protein (CP) and cysteine rich protein (CRP) genes. In the present study of CRPs to analyze their functions, we postulated that evolution of allexiviruses may have been largely directed by CRPs and thus proposed two evolutionary scenarios for allexiviruses based mainly on the presence or absence of IS and determined by how the allexiviruses challenge host resistance mechanisms (RNA silencing and autophagy). We found that both CP and CRP are RNA silencing suppressors (RSS), that they can inhibit each other’s RSS activity in the cytoplasm, and that CRP becomes a target of host autophagy in the cytoplasm but not CP. To mitigate CRP interference with CP, and to increase the CP’s RSS activity, allexiviruses developed two strategies: confinement of D-type CRP in the nucleus and degradation of I-type CRP by autophagy in the cytoplasm. Here, we demonstrate that viruses of the same genus achieve two completely different evolutionary scenarios by controlling expression and subcellular localization of CRP.

## Author summary

Allexiviruses in the genus *Allexivirus* mainly infect allium plants and are often found as mixed infections in garlic. The genus currently includes eight allexiviruses, with diverse sequence differences even among isolates within the same species. In plant viruses, RNA silencing suppressors (RSSs) play an important role in viral host adaptation by acting as virulence determinants, and have been found to be the driving force of viral evolution. The allexivirus protein, CRP has been found to be an RSS. The phylogenetic analysis based on this gene well supported the classification of the seven allexiviruses, and the same results were obtained using the coat protein (CP) gene. In addition, allexiviruses are divided into two groups depending on the presence or absence of an inserted sequence of 10 to 20 nucleotides between the *CP* and *CRP* genes. Because a balance between CP and CRP levels seemed to be important for viral pathogenicity, we investigated whether CP has some effect on the RSS activity of CRP, and found that CP was also an RSS. Surprisingly, we found that CP and CRP inhibit each other’s RSS activity; this is the first report on an antagonistic interaction between two RSSs. We here explain that allexiviruses have chosen two totally different evolutionary scenarios for their survival depending on how they maintain their RSS activity in infected cells.

## Introduction

The family *Alphaflexiviridae* consists of seven genera including plant and fungal viruses. Most of the viruses in *Alphaflexiviridae* have a positive-sense, single-stranded genomic RNA, which has five to seven open reading frames (ORFs) [1]. The genus *Allexivirus* includes eight viral species, *Garlic virus A*, *B*, *C*, *D*, *E* and *X* (GarV-A, GarV-B, GarV-C, GarV-D, GarV-E and GarV-X), *Shallot virus X* (ShVX) and *Garlic mite-borne filamentous virus*, that have a narrow host range limited to *Allium* species [2]. Allexiviruses commonly cause latent infection, but co-infection of garlic with potyviruses such as leek yellow stripe virus and onion yellow dwarf virus causes significant yield losses [3–5]. Allexiviruses have six ORFs. ORF1 encodes a viral replicase. The triple gene block (TGB1–3) proteins function in viral cell-to-cell or systemic movement [6–8]. ORF5 encodes the coat protein (CP), and ORF6 encodes the cysteine rich protein (CRP) [6,9].

Antiviral RNA silencing is a major resistance response in plants against viral infection. To evade host antiviral RNA silencing, most plant viruses have at least one RNA silencing suppressor (RSS). Some viruses have multiple RSSs, which target various steps in the antiviral RNA silencing pathway. Potyviruses encode multiple RSSs including P1, HC-Pro and VPg proteins [10–17], and rice stripe virus (RSV) has two RSSs, NS2 and NS3 proteins [18,19].

As a counterdefense against viral RSS proteins, plants use autophagy to degrade the RSS proteins. For example, selective autophagy mediated by NEIGHBOR OF BRCA1 (NBR1) targets HC-Pro of turnip mosaic virus (TuMV) [20]. The βC1 protein of a geminivirus satellite and the 2b protein of cucumber mosaic virus (CMV) are also degraded by autophagy [21,22]. Recently, Jiang et al. (2021) reported that the NbP3IP protein of *Nicotiana benthamiana* directs degradation of RSV NS3 via autophagy [23].

Viral CRPs have a nucleic acid-binding property and play multiple roles in viral infection [24]; they are known to suppress RNA silencing and to function as a pathogenicity determinant. For example, p14 of beet necrotic yellow vein virus and the 16K protein of tobacco rattle virus were reported as RSSs enhancing viral RNA levels in root [25,26]. The 19K protein of soil-borne wheat mosaic virus also serves as an RSS and functions as a pathogenicity determinant [27]. On the other hand, the cysteine-rich 8-kDa protein of potato mop-top virus (PMTV) has been reported to increase viral disease severity, but it does not have RSS activity [28]. In spite of the important roles of CRPs in viral pathogenicity, only a few studies on allexivirus CRPs have been reported. The ShVX CRP protein lacks RSS activity [29], but the CRP of GarV-X has weak RSS activity [9]. For CRPs of GarV-A and GarV-B, our group previously reported the effect of CRPs on potato virus X (PVX) accumulation and induction of necrotic symptoms when *CRP*s were expressed through the PVX vector [2]; their function as an RSS had not been demonstrated.

Compared to other plant viruses, allexiviruses have several unique features: (i) allexiviruses are often detected as mixed infections in the fields, (ii) they have an extremely narrow host range limited to *Allium* species, and (iii) their infection of garlic is permanently maintained throughout generations. They are classified into two groups; one group includes GarV-A, D and E, and the other group includes GarV-B, C and X [2,30,31,32,33]. The classification was based on two criteria: (1) deletion [D]-type, the stop codon for the *CP* ORF overlaps with the start codon of the *CRP* ORF; (2) insertion [I]-type, which has short inserted sequences (ISs) of varying lengths occur between the *CP* and *CRP* genes. Here, we demonstrate that the grouping of the six allexiviruses is linked to the presence or absence of the ISs between the *CP* and *CRP* genes [2]. In addition, the ISs were found to be TURBS (termination upstream ribosome-binding site)-like sequences, which may affect the recruiting of 18S rRNA to the start codon of CRP [2]. These findings suggest that the expression of *CRP* may play an important role in allexivirus evolution. We also compared the functions of the CRPs of allexiviruses GarV-B and GarV-D. Finally, we propose a model for how the two types of allexiviruses evolved defense against host antiviral responses (RNA silencing and autophagy) using the CRP as a driving force for evolution.

## Results

### Expression and subcellular localization of CP and CRP

**Fig 1:**
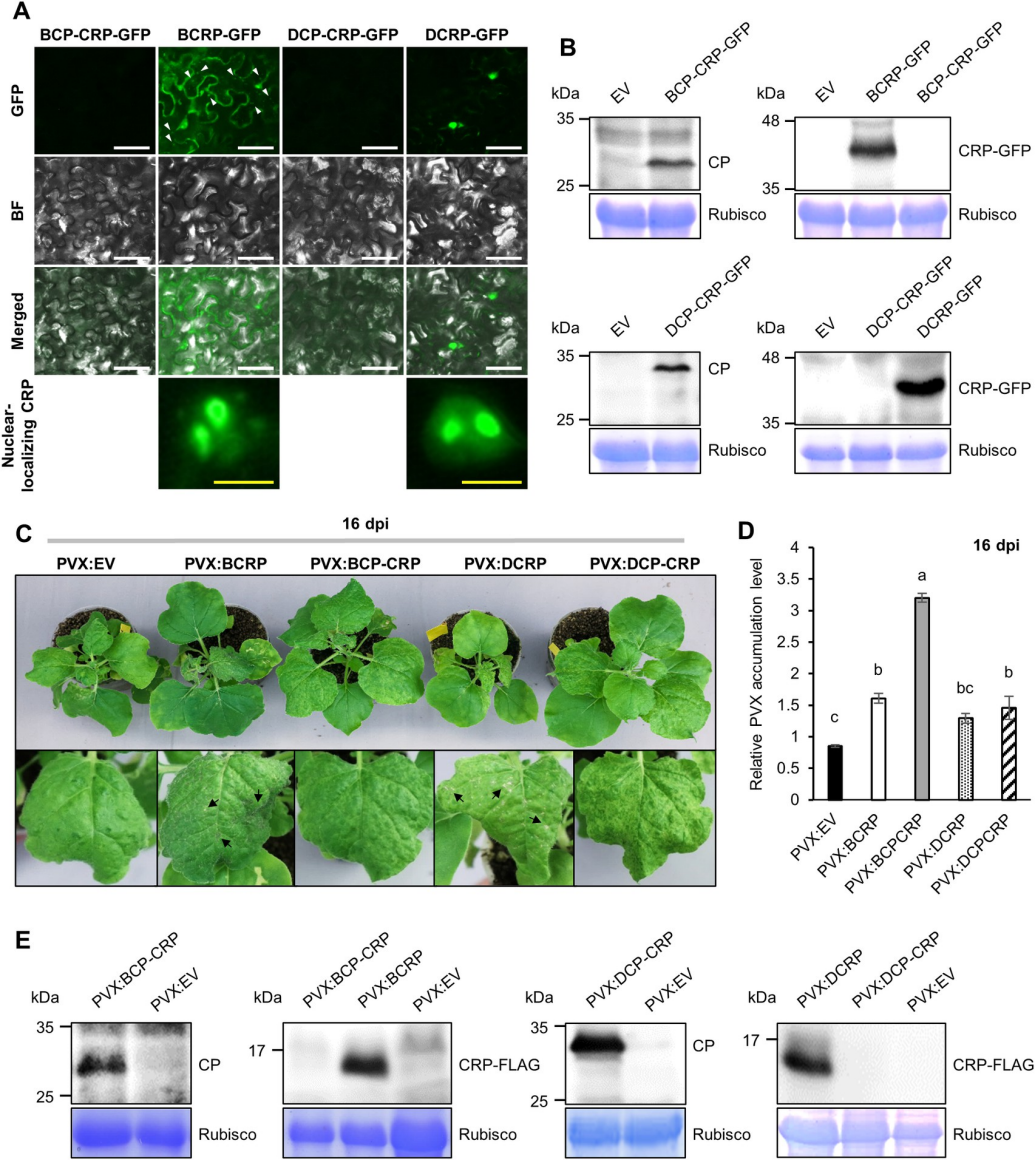
Expression of *CP* and *CRP* and their involvement in viral pathogenicity. (A) Fluorescence micrographs of GFP-fused CRPs of GarV-B and GarV-D in *N*. *benthamiana* leaves 2 days post agroinfiltration (dpa) with *Agrobacterium* resuspension cells carrying construct 35S:GarV-B-CP-CRP-GFP (BCP-CRP-GFP), 35S:GarV-B-CRP-GFP (BCRP-GFP), 35S:GarV-D-CP-CRP-GFP (DCP-CRP-GFP) or 35S:GarV-D-CRP-GFP (DCRP-GFP). GFP (GFP) and bright field (BF) images were taken. Scale bars: 50 μm (white), 10 μm (yellow). The representative small punta for BCRP-GFP are indicated by white arrowheads. (B) Western blots to detect allexivirus CPs and CRPs in *N*. *benthamiana* leaves expressing the CP-CRP or CRP construct. Total protein was extracted from leaf patch expressing BCP-CRP-GFP, BCRP-GFP, DCP-CRP-GFP or DCRP-GFP at 2 dpa. Anti-CP antibodies were used to detect CP and anti-GFP antibodies for CRP-GFP for GarV-B or GarV-D. Rubisco large subunit (Rubisco) was used as the loading control. (C) Symptoms on *N*. *benthamiana* plants infected with PVX expressing either CRP or CP-CRP at 16 days post inoculation (dpi). Necrotic spots on leaves of PVX:CRP-infected plants are indicated with black arrows. (D) Quantitative real-time RT-PCR to compare PVX RNA levels in the infected *N*. *benthamiana* plants at 16 dpi. Tukey’s multiple comparison test was used to compare means (±SEM) among individual plants (*n* = 3); different letters above bars indicate significant differences. (E) Western blot to detect CPs and CRP-FLAGs in *N*. *benthamiana* leaves infected with either PVX:CRP or PVX:CP-CRP. Anti-CP antibodies were used to detect CP and anti-FLAG monoclonal antibody for CRP-FLAG at 16 dpi. Rubisco large subunit (Rubisco) was used as the loading control.

In the present study, our phylogenetic tree inferred in a Bayesian framework also provided strong support for the allexivirus differentiation into the I- and D-types (S1 Fig). To compare *CRP* expression between those two types of allexiviruses, we selected GarV-B and GarV-D isolated from commercial garlic (S1 Fig) and cloned the sequences from the start codon of *CP* to the stop codon of *CRP* (CP-CRP) into the plant expression vector for *Agrobacterium*-mediated transient overexpression (S2 Fig). CRP was tagged with the green fluorescence protein (GFP) at its C-terminus, and *N*. *benthamiana* leaves that had been infiltrated with *Agrobacterium* carrying the CRP-GFP construct for GarV-B (BCRP-GFP) or for GarV-D (DCRP-GFP) were then observed for the GFP signal. BCRP was detected in the nucleus and cytoplasm, whereas DCRP was detected only in the nucleus (or nucleolus) (Fig 1A). These data suggest that the subcellular localization of CRPs may differ among allexiviruses. Unexpectedly, GFP fluorescence was not detected in leaves agroinfiltrated with the CP-CRP-GFP construct (BCP-CRP-GFP or DCP-CRP-GFP) where the *CP* and *CRP* sequences were not separated. To confirm the presence of CP and CRP, we performed western blot analysis. In accordance with the microscopic observations, CRP-GFP was not detected in the leaves agroinfiltrated with the CP-CRP-GFP constructs for either GarV-B or -D even though the *CP* was expressed in the same leaf tissues (Fig 1B). However, CRP-GFP proteins were detected in the leaf tissues agroinfiltrated with the CRP-GFP construct without the CP. These data suggest that the CRPs of GarV-B and GarV-D may not be efficiently translated from the mRNA for the CP-CRP sequence.

### Effect of expression of allexivirus *CP* and *CRP* on viral pathogenicity

To examine the role of CP and CRP in viral pathogenicity, we used the PVX vector, which belongs to *Alphaflexiviridae* family, to express allexivirus *CP* and *CRP* in *N*. *benthamiana*. PVX carrying the CP-CRP and *CRP* sequences (PVX:BCP-CRP, PVX:BCRP, PVX:DCP-CRP or PVX:DCRP) were created, then those recombinant PVXs were used separately to inoculate plants. At 16 days post inoculation (dpi), we found that all the recombinant PVXs expressing either *CP* or *CRP* induced more severe symptoms in *N*. *benthamiana* than the empty vector (PVX:EV) did (Fig 1C). Plants inoculated with either PVX:BCP-CRP or PVX:DCP-CRP showed also mosaic symptoms that were a little more severe than on plants inoculated with PVX:EV. Plants inoculated with either PVX:BCRP or PVX:DCRP showed the most severe leaf rolling and necrotic spots on the systemically infected leaves.

To quantify viral RNA levels in PVX-infected plants, we used real-time RT-PCRs with the PVX CP-specific primer pair at 16 dpi. As shown in Fig 1D, the viral RNA level was highest in the PVX:BCP-CRP-infected plants, and plants infected with either PVX:BCRP or PVX:DCP-CRP also had significantly higher levels of viral RNA than in PVX:EV-infected plants. The viral RNA level of PVX:DCRP was not significantly different from that of PVX:EV. Because *CRP* for the CP-CRP constructs did not seem to be expressed, CP must have played a role in increasing viral RNA accumulation. When the PVX vectors carrying the CP-CRP constructs were tested, only *CP* was expressed, similar to the results in Fig 1B, suggesting that the *CRP* gene in the CP-CRP constructs is not efficiently expressed (Fig 1E). *CRP* was actually expressed only from the PVX:BCRP and PVX:DCRP vectors.

**Fig 2:**
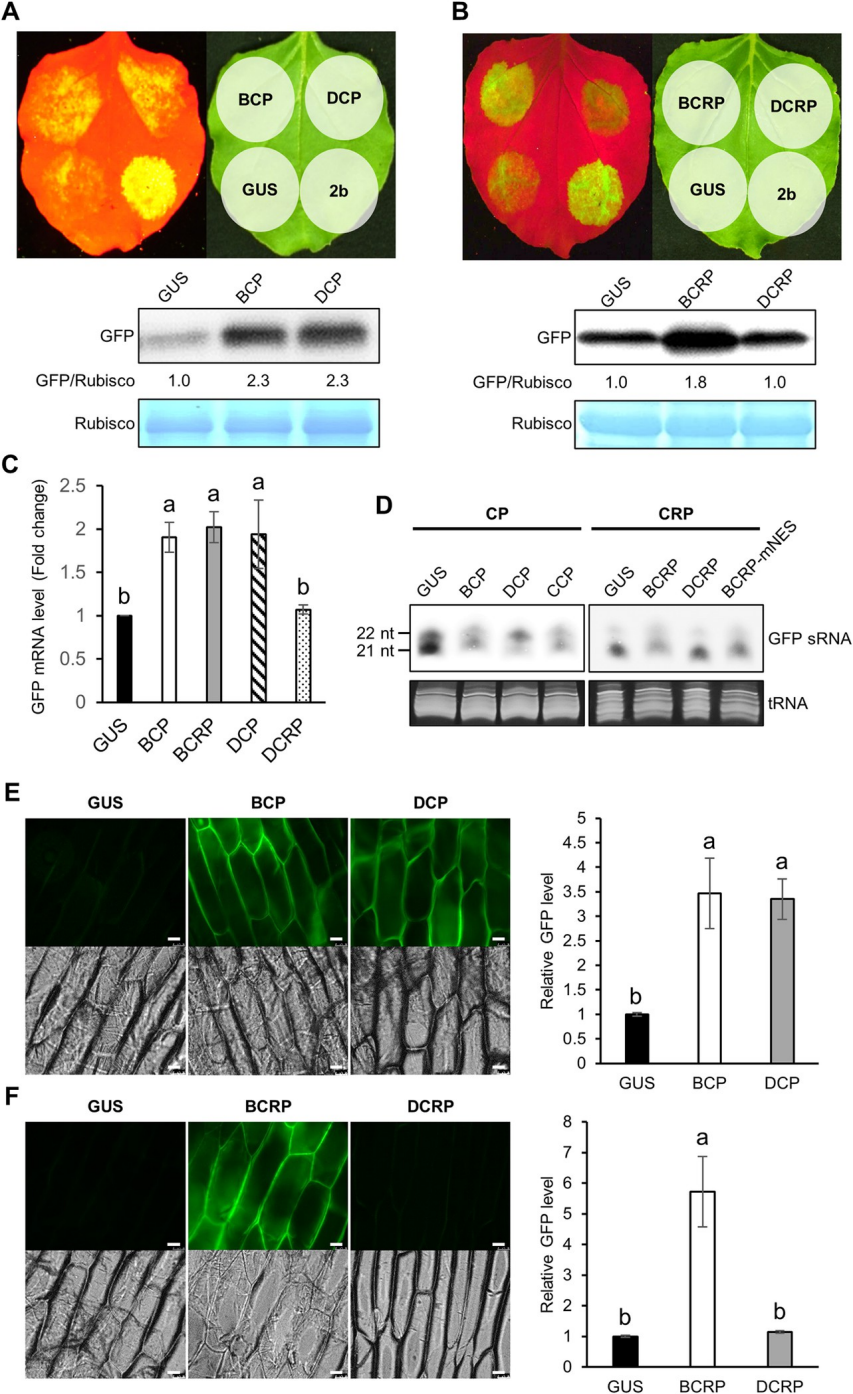
Comparison of RSS activity of CPs and CRPs from two allexiviruses. (A, B) RSS activity of CP and CRP in *N*. *benthamiana* leaves after co-infiltration with *Agrobacterium* cells containing either GFP and CP/CRP-FLAG at a 1:2 ratio. GUS, which was proven not to affect *GFP* silencing [34], was used as a negative control. Leaves viewed with UV light (left) and ambient light (right) at 5 dpa. Western blots using anti-GFP antibodies to compare GFP levels. The relative ratio of GFP/Rubisco was calculated after the value of GUS was set to 1.0. CBB-stained Rubisco large subunit (Rubisco) was included as a loading control. (C) Comparison of the *GFP* mRNA level between CP-expressing and CRP-expressing tissues by real-time RT-PCR. The agroinfiltrated patches, where *GFP* and *CP*/*CRP* had been co-expressed, were excised. Means (±SEM) of fold-changes (*n* = 3) are analyzed on log-transformed data by Tukey’s multiple comparison test (*P* < 0.05). Different letters above the bars indicate significant difference. (D) *GFP* sRNA accumulation in the CP- and CRP-expressing tissues used in Fig 2C. An antisense transcript of the *GFP* gene was used as a probe for northern blot analysis. An ethidium bromide (EtBr)-staining image of tRNA was included as a loading control. (E, F) RSS activity of CP and CRP in onion epidermal cells after infiltrated of scales with *Agrobacterium* resuspension carrying CP/CRP and GFP. GFP and bright field images were taken at 3 dpa using epifluorescence. Scale bars: 50 μm. Values for relative GFP expression were calculated using the LAS AF program. Mean (±SEM) fold changes (*n* = 9) were compared using Tukey’s multiple comparison test after log-transformed data, with the GUS (control) value set to 1.0 (*P* < 0.05).

Viral RSSs play an important role as a pathogenicity determinant by interfering with antiviral RNA silencing. For allexiviruses, GarV-X CRP was reported as an RSS, and the PVX that expressed the *CRP* enhanced PVX accumulation [9]. Because plants inoculated with the PVX vector expressing either *CP* or *CRP* showed severe symptoms and higher viral RNA levels compared to the control (Fig 1C and 1D), we then examined the RSS activity of the CP and CRP proteins. First, we analyzed RSS activity in *N*. *benthamiana* via a conventional agroinfiltration assay. The *GFP* gene was expressed with the *CP* or *CRP* gene by agroinfiltration in *N*. *benthamiana* leaves; we used *GUS* as a negative control because it had been previously proven not to affect GFP accumulation in our agroinfiltration assay [34]. As a result, the co-expression of GFP with GarV-B CP (BCP) or GarV-D CP (DCP) resulted in a stronger GFP signal in the agroinfiltrated patches than in the patches expressing the control (GFP+GUS), suggesting that both BCP and DCP have RSS activity (Fig 2A). For CRPs, BCRP had RSS activity with strong GFP fluorescence and a high GFP level, whereas DCRP did not suppress *GFP* silencing (Fig 2B). An increase in *GFP* mRNA level by BCP, DCP and BCRP in the agroinfiltrated tissues were further confirmed by real-time RT-PCR (Fig 2C). When *GFP* small RNAs (sRNAs) were detected by a northern blot, the sRNA accumulation levels were clearly lower in the BCP-, DCP-expressing leaf tissues than that in the control (GUS-expressing tissues) (Fig 2D). For CRP, although the sRNA accumulation level in the BCRP-expressing tissues was lower than that in the control, sRNAs accumulated in the DCRP-expressing tissues to the level similar to that in the control, consistent with the result of Fig 2B (Fig 2D). Therefore, the CRP RSS activity seemed to be associated with the sRNA levels, in good agreement with the previous report on GarV-X [9]. Because we have had experience with some viral RSSs functioning differently depending on the plant species used for the assay [35], we also examined RSS activity of the CPs and CRPs in onion. Consistent with the results of the assay using *N*. *benthamiana*, both BCP and DCP suppressed *GFP* silencing (Fig 2E), and BCRP but not DCRP also had RSS activity in onion cells (Fig 2F). Together, these results suggest that the allexivirus CPs and CRPs have RSS activity to counteract host antiviral RNA silencing, but only CRPs of some allexiviruses have RSS activity.

### Detection of putative sgRNAs for CRP expression

**Fig 3:**
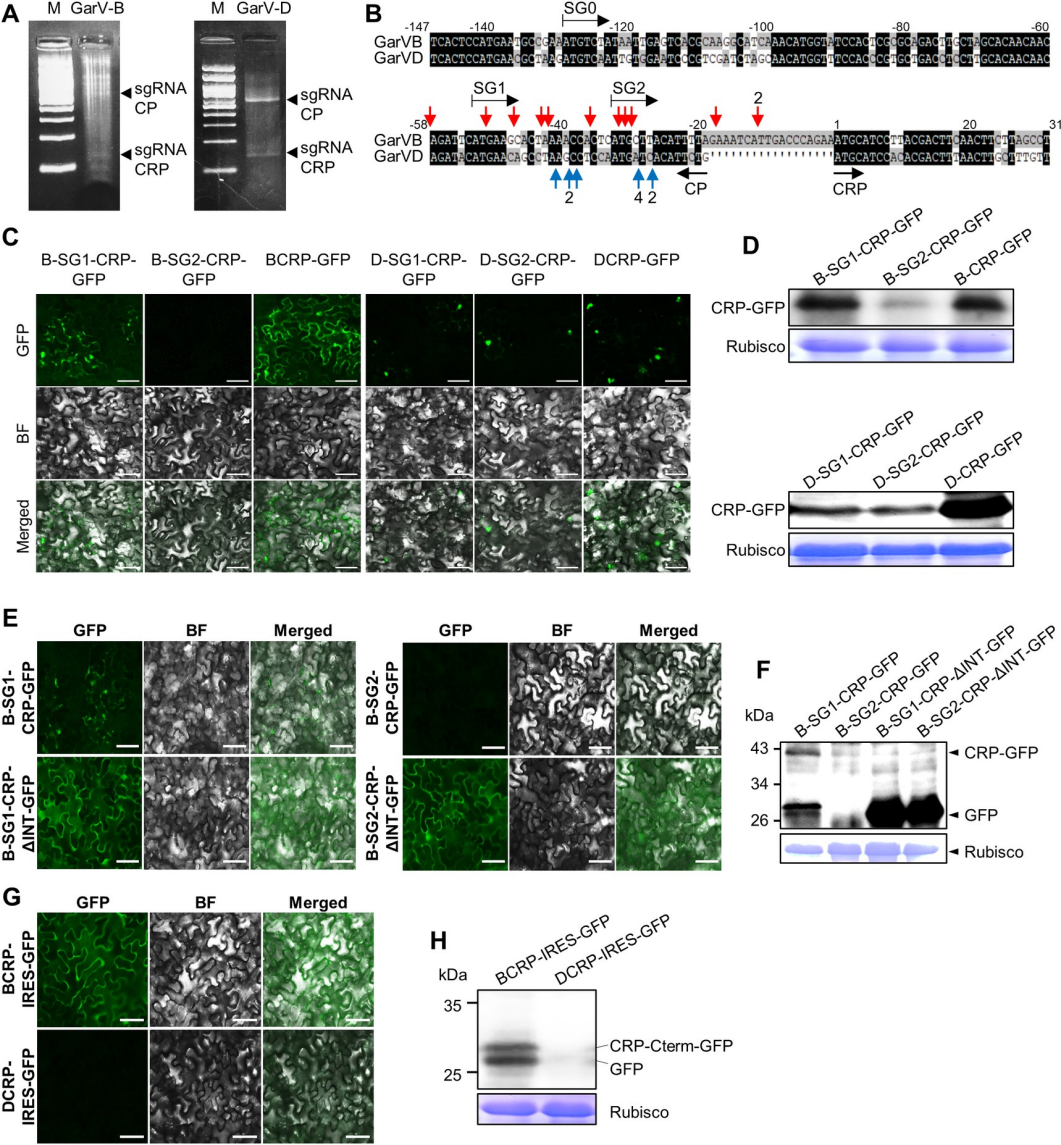
Detection of possible sgRNAs for allexivirus CRPs. (A) The 5′ RACE PCR products for possible sgRNAs of GarV-B and GarV-D in agarose gels. The black arrowheads indicate the PCR products corresponding to either subgenomic RNAs for CP (sgRNA-CP) or for sgRNA-CRP. Size marker (M): 1 kb DNA marker. (B) Map of the 5′ ends of the PCR products. Red and blue arrows indicate the start nucleotide of the PCR products of GarV-B and GarV-D, respectively. The numbers with the arrows indicate the number of sequences detected. The numbers above the alignment indicate relative nucleotide positions when the adenine residue in the start codon for the *CRP* gene was set to position 1. Based on the positions of the 5′ ends mapped on the viral sequence, we created three primers (SG0, SG1 and SG2) to generate sgRNA candidates. (C, D) Expression of CRP-GFP from the putative CRP sgRNAs. Suspensions of *Agrobacterium* carrying the constructs for the sgRNA candidates were used to infiltrate leaves of *N*. *benthamiana*. (C) Images of GFP fluorescence and bright fields (BF) taken at 2 dpa using epifluorescence. Scale bars: 50 μm. (D) Western blot with anti-GFP antibodies to detect BCRP- and DCRP-GFP in agroinfiltrated patches at 2 dpa. (E, F) Expression of B-SG1-CRP-ΔINT-GFP and B-SG2-CRP-ΔINT-GFP in agroinfiltrated patches that lack the IS sequences between *CP* and *CRP* of GarV-B from the sgRNA candidates (S2 Fig). (E) Images of GFP fluorescence and BF taken at 2 dpa using epifluorescence. Scale bars: 50 μm. (F) Western blot with anti-GFP antibodies to detect CRP-GFP at 2 dpa. (G, H) Expression of the GarV-B *CRP* ORF from the CRP-IRES-GFP constructs in agroinfiltrated patches at 2 dpa. The 3′ end of the *CRP* gene (81 nt for GarV-B, 84 nt for GarV-D) including the IRES sequence was fused to the *GFP* gene, and used to agroinfiltrate *N*. *benthamiana* leaves. Scale bars: 50 μm. (H) Western blot with anti-GFP antibodies to detect the GFP bands. CBB-stained images of Rubisco large subunit are shown as a loading control (D, F, H).

Because the *CRP* genes of GarV-B and GarV-D were expressed from neither the CP-CRP construct in the pBE2113 vector via agroinfiltration nor that in PVX vector, we anticipated that the *CRP* genes might be expressed from subgenomic RNAs (sgRNAs). To detect sgRNAs, which should start in the middle of the *CP* ORF, we used 5′ RACE (5′ rapid amplification of cDNA end) to identify the 5′ ends of sgRNAs (S3 Fig). Total RNA extracted from GarV-B- and GarV-D-infected garlic plants were used as templates to detect the 5′ end of viral sgRNAs. The amplicons of the 5′ RACE (Fig 3A) were sequenced to identify the sgRNAs. As shown in Fig 3B, we found that several RNA fragments actually started upstream of the *CRP* genes; we hereafter call them sgRNA-CRPs for convenience. The longest sgRNA-CRPs for GarV-B and GarV-D started around 58 and 22 bases, respectively, upstream of the start codon of the *CRP* genes (Fig 3B). To examine whether the *CRP* genes are expressed from those sgRNA-CRPs, GFP was fused to the C-terminal of CRP. We then prepared three primers mimicking the 5′ ends of sgRNAs (Fig 3B; SG0, SG1 and SG2) and the primer for the 3′ end of the *GFP* gene. Using those primers, we amplified the PCR products that contained the various sgRNA-CRP-GFP construct, cloned the amplicons in the plant expression vector and expressed the constructs via agroinfiltration in *N*. *benthamiana* leaves. As shown in Fig 3C, the GFP signal was observed in the cytoplasm/nucleus in the patches agroinfiltrated with B-SG1-CRP-GFP, but not in the patches with B-SG2-CRP-GFP, suggesting that GarV-B sgRNA-CRP may start upstream of SG1. On the other hand, the leaves infiltrated with either D-SG1-CRP-GFP or D-SG2-CRP-GFP had GFP signal only in the nucleus. Our western blot analysis to detect CRP-GFP revealed that the level of CRP-GFP expressed from B-SG1-CRP-GFP was much higher than that from B-SG2-CRP-GFP (Fig 3D). The CRP levels were similar between D-SG1-CRP-GFP and D-SG2-CRP-GFP. These observations support that *CRP* is expressed from those putative sgRNAs, starting inside the 3′ region of the *CP* gene. Meanwhile, because we could not detect *CRP* expression from the sgRNA candidate that started at SG0, we inferred that the sgRNA-CRPs of the two allexiviruses would start between SG0 and SG1 (Fig 3B). To further confirm the presence of sgRNA-CRPs, we analyzed whether the sgRNAs are encapsidated in the viral particles. We first isolated allexivirus particles from infected garlic tissues by immunoprecipitation (S4A Fig); this time, GarV-X was used instead of GarV-B because we were not able to obtain garlic bulbs that had been infected with both GarV-B and GarV-D. As shown in S4B Fig, the specific precipitation of allexivirus particles was confirmed by immunocapture RT-PCR. We then performed 5′ RACE using the RNAs isolated from the purified viruses as illustrated in S4C Fig. As a result of sequencing analyses (S4D Fig), we obtained a similar result to that in Fig 3B; allexiviruses thus seemed to encapsidate viral subgenomic RNAs in their particles.

### Inserted sequences between *CP* and *CRP* are necessary for the GarV-B CRP expression

Are the ISs between *CP* and *CRP* of I-type really necessary for the *CRP* expression? In our previous paper, we discussed the possibility that the ISs may play a role as a ribosomal RNA binding site [2]. To examine this possibility, we created the sgRNA-CRP constructs that lack ISs between *CP* and *CRP* of GarV-B (S2 Fig, B-SG1-CRP-ΔINT-GFP and B-SG2-CRP-ΔINT-GFP). The constructs were then transiently expressed in *N*. *benthamiana* by agroinfiltration. Unexpectedly, the GFP signal was much stronger in cells expressing B-SG1-CRP-ΔINT-GFP or B-SG2-CRP-ΔINT-GFP than in cells expressing B-SG1-CRP-GFP (Fig 3E). In addition, a western blot analysis using anti-GFP antibody showed a strong band corresponding to the GFP (~29 kDa) in leaves that expressed B-SG1-CRP-ΔINT-GFP or B-SG2-CRP-ΔINT-GFP (Fig 3F) and in leaves that expressed B-SG1-CRP-GFP. We thus assumed that GarV-B may have a strong internal ribosome entry site (IRES) inside of the *CRP* ORF, which might compete for ribosome binding with the ISs upstream of the *CRP* gene for CRP translation. To examine this idea, we created the constructs where the *CRP* 3′ end region (~100 bp) was fused to the 5′ end of the *GFP* gene to generate the IRES:GFP constructs (S2 Fig, BCRP-IRES-GFP and DCRP-IRES-GFP). When the constructs were expressed in *N*. *benthamiana* after agroinfiltration, the GFP signal was strong in leaves expressing BCRP-IRES-GFP but not in the leaves expressing DCRP-IRES-GFP (Fig 3G). GFP and GFP fused to a protein translated from an AUG in the C-terminal of BCRP (S5 Fig) were detected in leaves expressing BCRP-IRES-GFP (Fig 3H). These results indicate the presence of IRES in the GarV-B *CRP* ORF that can compete for ribosome binding with the ISs upstream of the *CRP* gene.

### GarV-B CRP contains a nuclear export signal (NES) required for RSS activity

As previously reported by Zhang et al. (2018) [9], GarV-X CRP has a nuclear localization signal (NLS) motif, which is crucial for localization in the nucleus/nucleolus and suppression of RNA silencing. When we aligned six allexiviruses (GarV-A–E and X), we found that the NLS motif is conserved in all the CRPs (S6A Fig), suggesting that all allexivirus CRPs are imported into the nucleus. However, as shown in Fig 1A, their subcellular location was totally different between GarV-B and GarV-D. GarV-B CRP was localized in the nucleus and cytoplasm, whereas GarV-D CRP was limited to the nucleus. We then wondered whether GarV-B CRP might contain a functional nuclear export signal (NES) motif(s) and used the program NetNES 1.1 to search for such motifs. All CRPs of the I-type allexiviruses (GarV-B, C and X), which contain ISs between *CP* and *CRP*, had a putative NES motif (LXXLXL or LXXXXLXL) from L115 to L122 (S6B Fig), but the CRPs of the D-type (GarV-A, D and E) did not. In addition, the NES motif in CRP of I-type was found to partially overlap the possible IRES region we identified inside *CRP* (S5 and S6A Figs).

**Fig 4:**
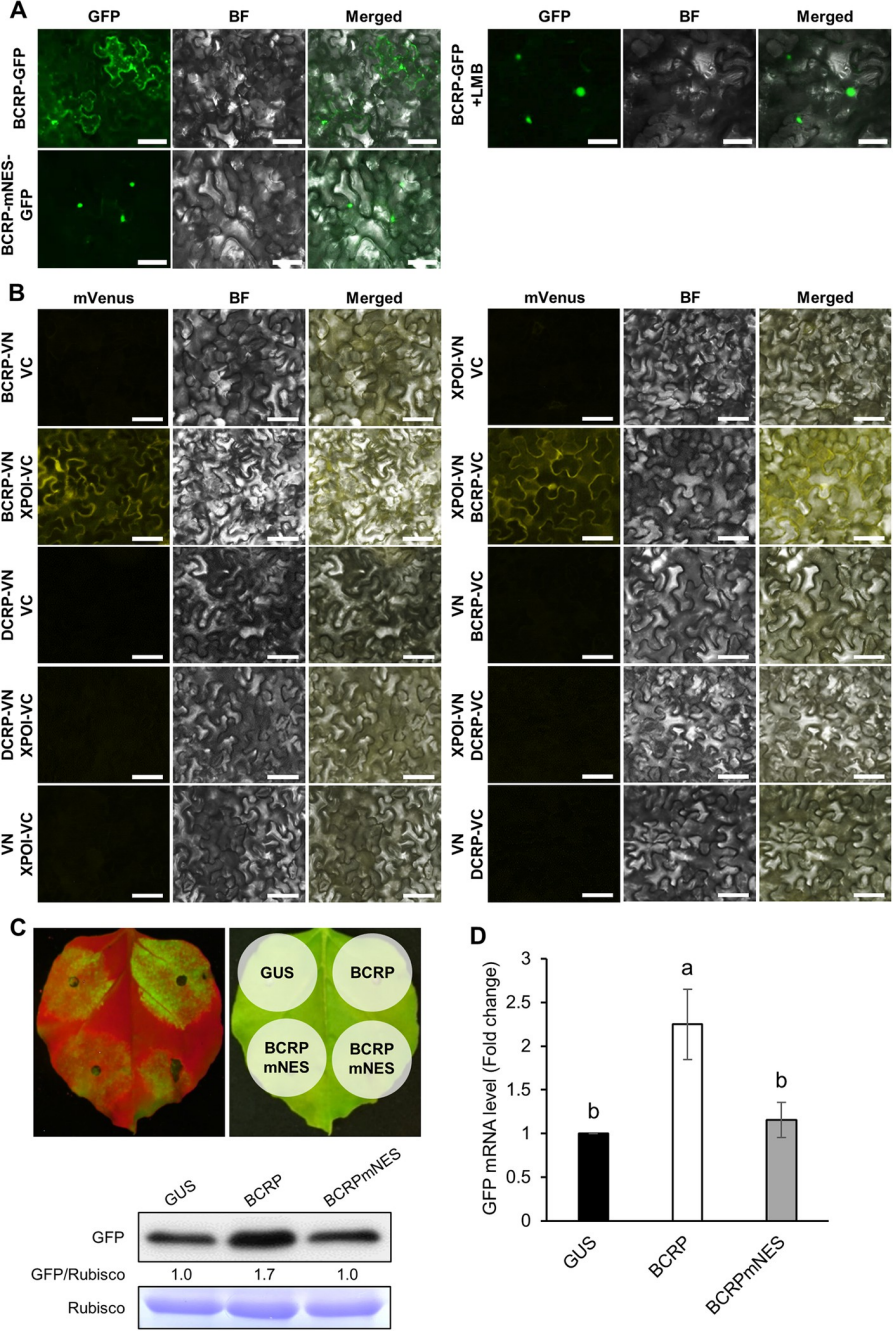
GarV-B CRP with an NES for nuclear export and its RSS activity. (A) Effect of the NES mutation or LMB treatment on the GarV-B CRP’s intracellular localization. *N*. *benthamiana* leaves were agroinfiltrated with GFP-tagged BCRP and its NES mutant (BCRP-mNES). LMB (40 nM) was applied at 2 dpa. GFP (GFP) and bright field (BF) images were taken; merged images of GFP and BF are shown. Scale bars: 50 μm. (B) BiFC assay to detect any interaction between CRP and XPOI. Either the N- or C-terminal region of the mVenus fluorescence protein (VN or VC) was fused to CRPs and XPOI, and *N*. *benthamiana* leaves were agroinfiltrated with the fusion proteins. The images were taken at 2 dpa. Scale bars: 50 μm. (C, D) Effect of the NES mutation on RSS activity of GarV-B CRP. *Agrobacterium* resuspension cells containing the GFP and CP/CRP-FLAG constructs were used at a 1:2 ratio for co-infiltration. (C) Images were taken under UV light at 5 dpa, and then the agroinfiltrated patches were directly excised for western blot analyses using anti-GFP antibodies to compare the GFP levels. The relative ratio of GFP/Rubisco was calculated after the value of GUS (control) was set to 1.0. CBB-stained Rubisco large subunit (Rubisco) is shown as a loading control. Epifluorescence was used for all fluorescent images. (D) The same tissues were used for real-time RT-PCR to compare the *GFP* mRNA levels as described in Fig 2C (*n* = 3 or 4).

To determine whether the predicted NES actually functions in nuclear export of CRP, we generated a mutant by replacing the NES region of BCRP with the corresponding region of DCRP (BCRP-mNES), and GFP was fused to the CRPs to observe subcellular localization. As expected, the BCRP-mNES was localized in the nucleus (Fig 4A), while its accumulation level was comparable to that of BCRP (S7 Fig). Because the NES motifs are generally recognized by exportin (XPOI), we then tested whether XPOI-mediated nuclear export of GarV-B CRP was involved using leptomycin B (LMB), an inhibitor of XPOI. Similar to BCRP-mNES, the BCRP in the LMB-treated tissues was greatly concentrated in the nucleus compared with the nontreated tissues (Fig 4A), suggesting that the nuclear export of BCRP is mediated by XPOI. We then used a bimolecular fluorescence complementation (BiFC) assay to test for a direct *in vivo* interaction between CRP and XPOI. The N- or C-terminal region of mVenus (VN or VC) was fused at the C-terminal of BCRP, DCRP and XPOI, then the fusion proteins were used to agroinfiltrate *N*. *benthamiana* for co-expression of the proteins. As shown in Fig 4B, we detected an interaction between BCRP and XPOI in the reciprocal combination, but not between DCRP and XPOI. These results suggest that the interaction with XPOI is required for the nuclear export of the CRPs.

We then wondered whether the BCRP-mNES has RSS activity, considering that DCRP lacks RSS activity (Fig 2). When RSS activity of the two constructs (BCRP and BCRP-mNES) was compared, the GFP expression level in the patch infiltrated with BCRP-mNES was similar to that of the patch infiltrated with the negative control expressing GUS (Fig 4C and 4D), despite of the fact that BCRP-mNES accumulated at a level similar to BCRP (S7 Fig). In addition, the accumulation level of *GFP* sRNA was actually increased in the BCRP-mNES-expressing tissues (Fig 2D), suggesting that BCRP-mNES is not functioning as an RSS. We further tested whether an insertion of BCRP’s NES into DCRP can enhance the cytoplasmic accumulation and RSS activity of DCRP. As a result, the NES-inserted DCRP (DCRP-NES) accumulated abundantly in the cytoplasm (S8A Fig) and showed distinct RSS activity in contrast to the original DCRP (S8B and S8C Fig). These results suggest that the cytoplasmic CRPs may have a primary role in the suppression of RNA silencing and that its NES-mediated nuclear export is important for its RSS activity.

### Autophagy-mediated protein degradation affects stability and RSS activity of GarV-B CRP

**Fig 5:**
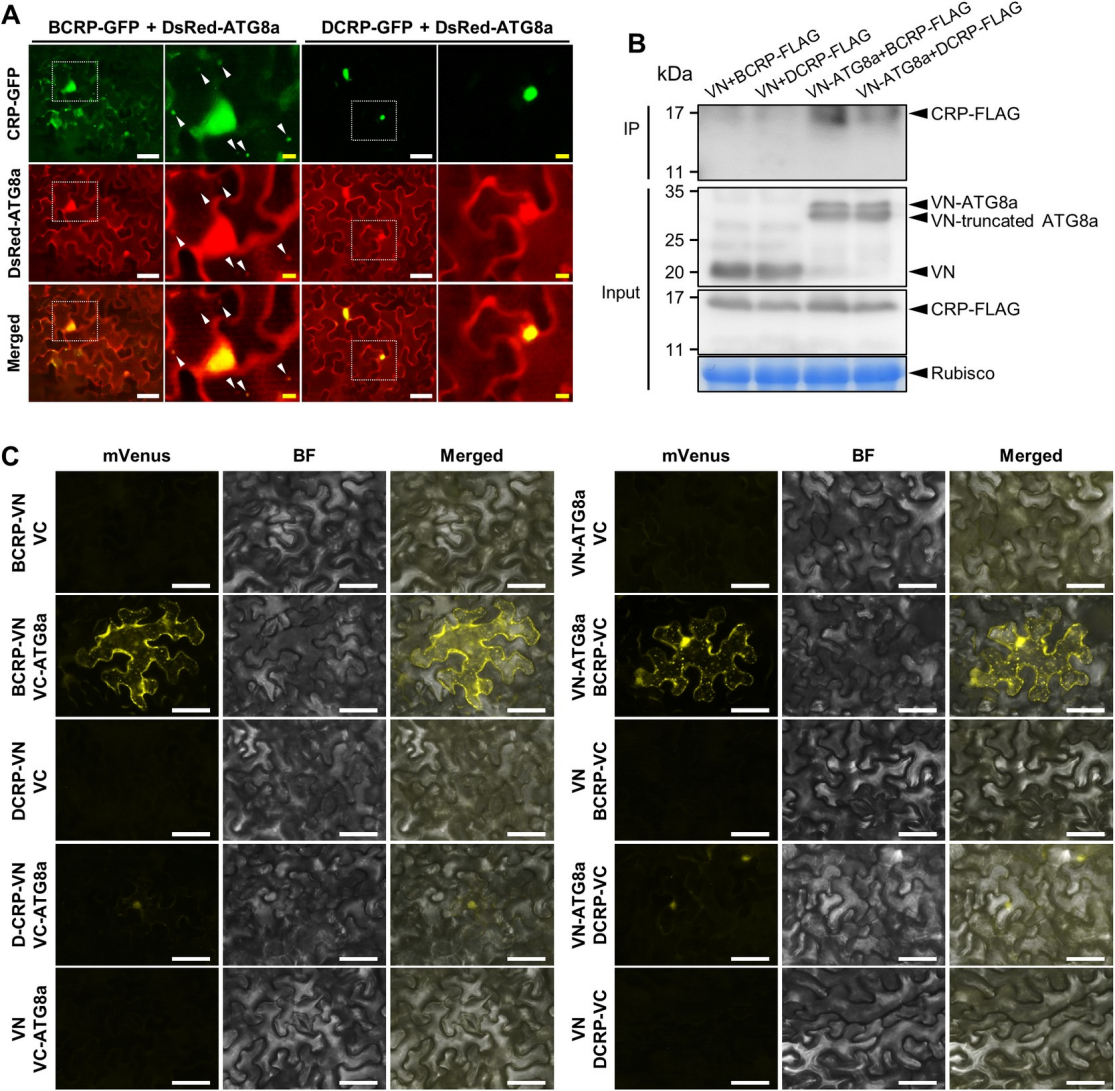
CRPs of GarV-B and GarV-D interact with ATG8a. (A) Co-localization of CRPs and ATG8a. CRP-GFP and DsRed-ATG8a were co-expressed in *N*. *benthamiana* leaves via agroinfiltration. GFP and DsRed fluorescence was observed using epifluorescence. The images on the right side are the close-up images of the white-dotted areas on the left side. Autophagosome-like vesicles are indicated by white arrowheads in the images of [BCRP-GFP+DsRed-ATG8a]. Scale bars: 50 μm (white) and 10 μm (yellow). (B) *In vivo* co-immunoprecipitation of CRPs and ATG8a. The CRP-FLAG/VN-ATG8a complexes were co-immunoprecipitated using anti-GFP (anti-mVenus) antibodies, and CRP-FLAGs in the precipitant were detected by western blot analysis using an anti-FLAG antibody. VN was used as a control. The CRP-FLAGs and VN-ATG8a in the total protein extracts were detected as inputs. CBB-stained Rubisco large subunit (Rubisco) is shown as a loading control. (C) BiFC assay to detect an interaction between CRP and ATG8a. BCRP, DCRP and ATG8a were fused to either the N- or C-terminal of mVenus, and the constructs for the fusion proteins were used to agroinfiltrate *N*. *benthamiana* leaves to co-express the two proteins. The mVenus fluorescence was observed at 2 dpa using an epifluorescence microscope. Scale bars: 50 μm.

**Fig 6:**
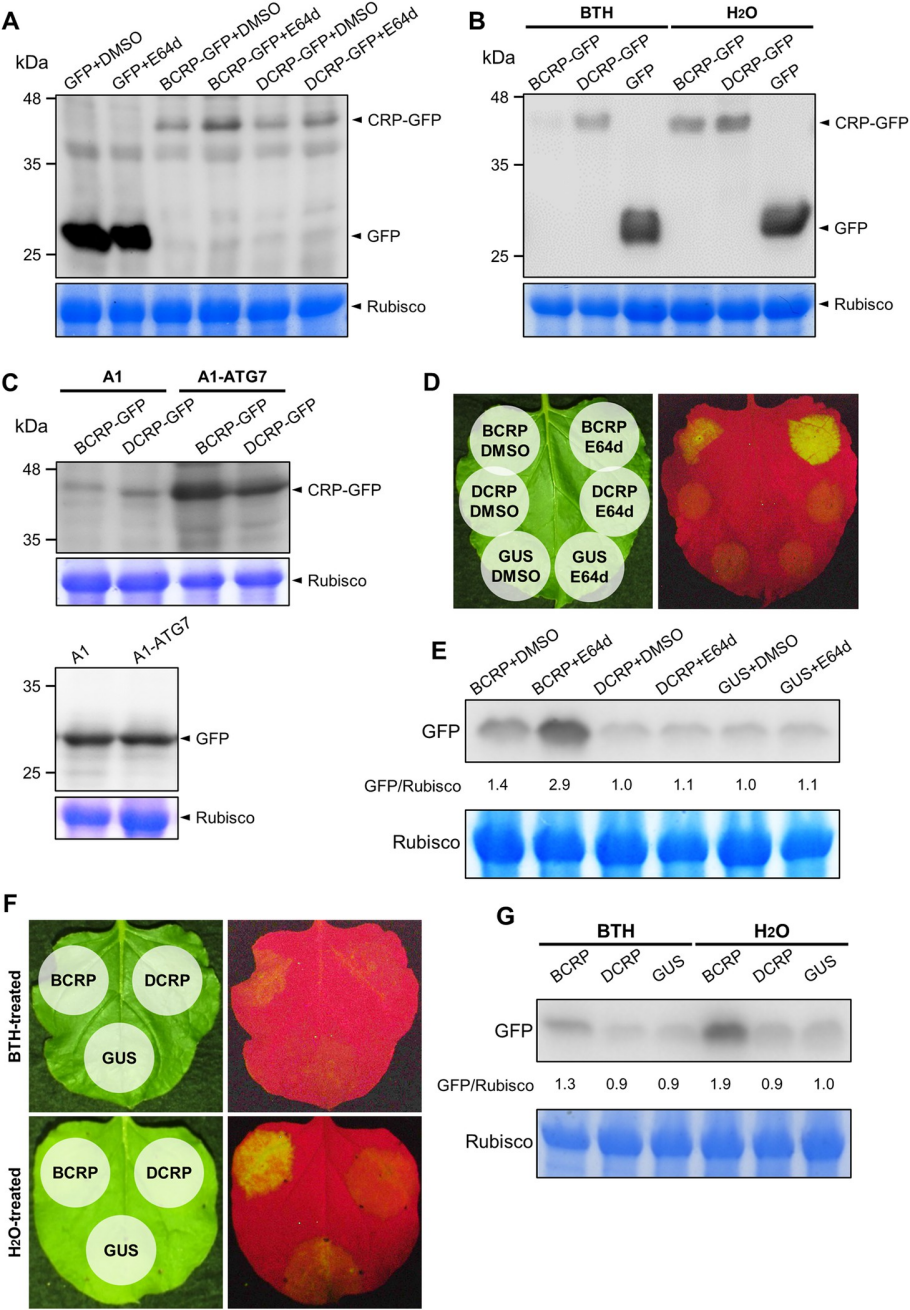
Effect of cellular autophagy on CRP accumulation and CRP RSS activity. (A, B) Effect of E64d or BTH on CRP accumulation. (A) At 24 h post agroinfiltration (hpa) with the CRP-GFP and the empty GFP constructs, 20 μM E64d was used to infiltrate the agroinfiltrated patches where GFP-fused CRP had been previously expressed. DMSO (2%), the solvent for E64d was used as a negative control. (B) For the BTH treatment, BTH (1 mM) was sprayed over the agroinfiltrated leaves at 24 hpa. The E64d- or BTH-treated tissues were excised at 16 h after treatment, and CRP-GFP was detected by western blotting. CBB-stained Rubisco large subunit (Rubisco) is shown as a loading control. (C) Effect of *ATG7* silencing on CRP accumulation. *N*. *benthamiana ATG7* silencing was conducted by the CMV VIGS system (S11 Fig), and then the CRP-GFP genes were expressed in plants by agroinfiltration at 14 dpi. The A1 vector was used as an empty vector control. The CRP accumulation levels were evaluated by western blot analysis at 3 dpa. (D-G) *N*. *benthamiana* leaves were co-infiltrated with *Agrobacterium* cells carrying GFP and CRP-FLAG in a 1:2 ratio, then at 24 hpa, the agroinfiltrated patches were infiltrated with 20 μM E64d (D, E) or sprayed with 1 mM BTH (F, G). DMSO (2%) and H_2_O were used as negative controls. (D, F) The intensity of GFP fluorescence was compared among the patches treated with E64d, BTH and the nontreated control at 5 dpa. (E, G) Each patch was excised, and the GFP levels were estimated by western blot analysis. The relative ratio of GFP/Rubisco was calculated after the value of GUS was set to 1.0. CBB-stained Rubisco large subunit (Rubisco) is shown as a loading control.

When we investigated the intracellular localization of the GFP-fused CRPs, we found that BCRP was localized at numerous puncta representing possibly autophagosomes in the cytosol, which were not detected when DCRP was expressed (Fig 1A). To determine whether the CRPs can interact with a factor(s) such as ATG8 in autophagy, we first tested for co-localization of CRPs and ATG8a, an autophagy marker by agroinfiltrating *N*. *benthamiana* leaves with the CRP-GFP fusion protein and DsRed-ATG8a. As shown in Fig 5A, BCRP and ATG8a co-localized in the nucleus and the cytoplasm, and the signal was especially strong in the small autophagosome-like vesicles. DCRP and ATG8a, however, co-localized only in the nucleus. We then tested the possibility that BCP or DCP can also induce autophagosomes. When CP and DsRed-ATG8a were co-expressed in *N*. *benthamiana* leaves via agroinfiltration, we did not find any puncta as observed in Fig 5A, which represent autophagosomes (S9 Fig); this result thus suggests that the CPs of GarV-B and GarV-D will not be targeted by autophagy. To confirm the interaction between CRP and ATG8a, we then performed *in vivo* co-imunoprecipitation (co-IP) for the FLAG-tagged CRPs and VN-ATG8a. As a result, CRP-FLAG/VN-ATG8a complexes were specifically precipitated using anti-GFP (anti-mVenus) antibodies, which can recognize the VN portion (Fig 5B). We found that both BCRP and DCRP could be precipitated with ATG8a, and that a higher level of BCRP was detected in the precipitant than that of DCRP, suggesting that BCRP must be more abundantly associated with ATG8a than DCRP (Fig 5B). The interaction between CRPs and ATG8a was further confirmed by a BiFC assay. As shown in Fig 5C, BCRP interacted with ATG8a in a reciprocal manner, and the interaction was observed as strong mVenus signals in the cytoplasmic autophagosomes. For DCRP and ATG8a, we could detect only weak fluorescence signals in the nucleus. These results support that BCRP rather than DCRP is much strongly associated with a factor(s) in autophagy, indicating that the CRPs are degraded via autophagy in the cytosol. To further confirm whether the CRPs interact with a factor(s) in autophagy, we treated the agroinfiltrated tissues with 20 μM of E64d, an autophagy inhibitor, to examine its effect on the CRP levels. Our western blot analyses showed that the BCRP level was much higher than in the nontreated control, but the level of DCRP was less affected by the E64d treatment (Fig 6A). Similarly, the effect of E64d on the BCRP or BCRP-mNES accumulation showed that BCRP accumulated at a significantly higher level by E64d than BCRP-mNES, suggesting that the nuclear-localizing CRPs may be less affected by autophagy (S10 Fig). In contrast, when we treated the tissues with 1 mM of BTH to induce autophagy, BCRP accumulation was strongly inhibited and DCRP accumulation was slightly repressed compared with the nontreated (Fig 6B). To further confirm whether the accumulation levels of CRPs are indeed controlled by autophagy, we downregulated the *ATG7* gene by virus-induced gene silencing (VIGS) because it has been previously reported that VIGS of *N*. *bentamiana ATG7* resulted in impaired autophagy [36]. We first created the recombinant CMV-A1 vector carrying a partial *ATG7* sequence (190-nt, A1-ATG7) (S11A Fig). The transcripts of CMV RNA1, RNA3 and the recombinant A1 vector were then inoculated to *N*. *benthamiana* plants, and at 14 dpi, the CRP-GFP genes were expressed in systemically infected upper leaves by agroinfiltration. At 3 days post agroinfiltration (dpa), the accumulation levels of CRPs were evaluated by western blot analysis. As shown in S11 Fig, we found that the level of *ATG7* mRNA in A1-ATG7-infected plants was reduced almost half of that in the control (A1-infected plants). As expected, the accumulation levels of both CRPs were greatly enhanced by *ATG7* VIGS; the BCRP level was a little higher than that of DCRP (Fig 6C).

We next examined the effect of autophagy on CRP’s RSS activity first by agroinfiltrating leaves with either BCRP or DCRP with GFP, then 24 h later by treating the agroinfiltrated patches with 20 μM E64d or 1 mM BTH. After 4 days, the RSS activity of BCRP greatly increased compared with the untreated control but DCRP did not affect the *GFP* expression with or without E64d (Fig 6D and 6E). In contrast, BTH reduced the RSS activity of BCRP but that of DCRP was not affected (Fig 6F and 6G). Therefore, these results suggest that the RSS activity of BCRP is repressed through autophagy while autophagy has only a minor effect on DCRP.

### Antagonistic interactions between CP and CRP in RSS activity and viral pathogenicity

**Fig 7:**
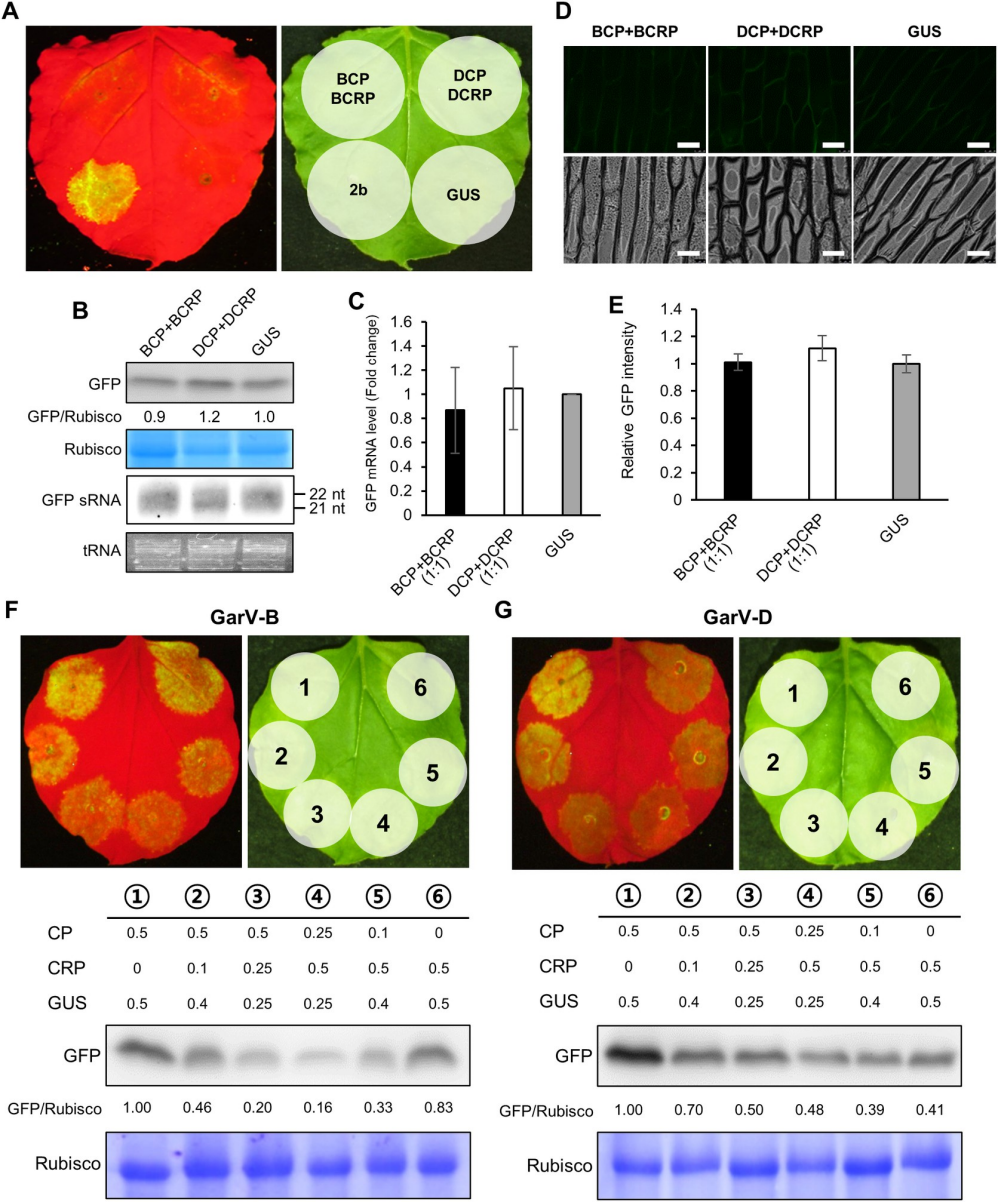
Antagonistic interactions between CP and CRP in overall RSS activity when the proteins are co-expressed. (A-C) Effect of co-expression of CP and CRP on overall RSS activity. *N*. *benthamiana* leaves were co-infiltrated with *Agrobacterium* carrying the GFP, CP and CRP-FLAG constructs at a ratio of 1:1:1. CMV 2b was used as the positive control and GUS as the negative. (A) GFP fluorescence images at 5 dpa under UV light (left) and natural light (right). (B) Western blot and northern blot analyses were performed to detect GFP and *GFP* sRNAs, respectively, in *N*. *benthamiana* leaves co-infiltrated with *Agrobacterium* carrying GFP, CP and CRP at 5 dpa. The relative ratio of GFP/Rubisco was calculated with the value of GUS set to 1.0. (C) The same tissues were used for real-time RT-PCR to compare the *GFP* mRNA levels as described in Fig 2C (*n* = 5). (D, E) Antagonistic effect of co-expression of CP and CRP on overall RSS activity in onion epidermal cells. Images in (D) were taken under UV (top row) and bright field (bottom row) at 3 dpa. (E) Relative GFP fluorescence intensities measured using the LAS AF software (Leica) and calculated as fold-changes with the value of GUS set to 1.0. Mean (±SEM) fold-change values (*n* = 7) were log-transformed and analyzed for significant differences using Tukey’s multiple comparison test. (F, G) Antagonistic interactions between CP and CRP in RSS activity observed when the expression ratio of *CP* to *CRP* was gradually changed. Overall RSS activity was examined in agroinfiltration patches expressing the constructs [GarV-B CP + GarV-B CRP] (F) and [GarV-D CP + GarV-D CRP] (G) at various *CP*/*CRP* expression ratios. Bacterial concentrations used to infiltrate the patches are given as OD_600_ values. GFP levels were determined using western blots. The relative ratio of GFP/Rubisco is given below the western blot. The CBB-stained Rubisco large subunit (Rubisco) is shown as a loading control (B, F, G).

Our results demonstrated that BCP, DCP and BCRP can serve as RSSs (Fig 2). To determine whether the redundant roles of CP and CRP in suppressing RNA silencing are synergetic or antagonistic, we examined their RSS activity in *N*. *benthamiana* when the two proteins were co-expressed. As a result, GFP fluorescence and the accumulation levels of *GFP* mRNA were similar in the agroinfiltrated tissues expressing both CP and CRP to those in GUS-expressing tissues regardless of whether the two proteins were derived from GarV-B or GarV-D (Fig 7A–7C). When both *CP* and *CRP* were simultaneously expressed, the *GFP* sRNA levels in all the tested tissues were detected to the levels similar to that in the control (*GUS*), indicating that no RSS activity was observed in the presence of both CP and CRP (Fig 7B), and that CP and CRP appear to inhibit each other’s RSS activity. RSS activity was also repressed in onion cells after the co-expression of CP and CRP (Fig 7D and 7E). We then examined the localization of CRP in the presence of CP, and found that the localization of BCRP and DCRP did not change (S12 Fig). During allexivirus infection, the level of CP gradually increased while the cytoplasmic CRPs underwent autophagy-mediated degradation. Thus, we tested how the co-expression of different ratios of CP and CRP alters overall RSS activity and found that the total RSS activity decreased as the CRP levels increased (Fig 7F and 7G), regardless of GarV-B or GarV-D. When CP and CRP were co-expressed in our agroinfiltration system, the level of each protein accumulation simply depended on the amount of the infiltrated *Agrobacterium* (S13A and S13B Fig). However, the antagonistic interaction between the CRP and CP of GarV-D seemed to be weaker than between the CRP and CP of GarV-B.

CRP and CP of allexivirus have RSS activity, but when the two proteins accumulate simultaneously in the cells, they inhibit each other’s RSS activity. As a first step to elucidate this mechanism, the interaction between the two proteins was analyzed by a co-IP assay. The allexivirus CPs are approximately 27 kDa in size. However, western blot analyses using anti-CP antibodies revealed a major band of BCP to be 27 kDa with a minor band around 32 kDa, while only a band with 32 kDa was detected for DCP (S14 Fig). This observation suggests that the allexivirus CPs are subjected to some posttranslational modification, and the modification patterns are different among allexiviruses. By the co-IP assay, we could detect some weak binding between CRP and the modified CP (S14 Fig). This suggests that there would be at least a weak affinity between the two proteins, and that the possible interaction may affect their respective RSS activities.

**Fig 8:**
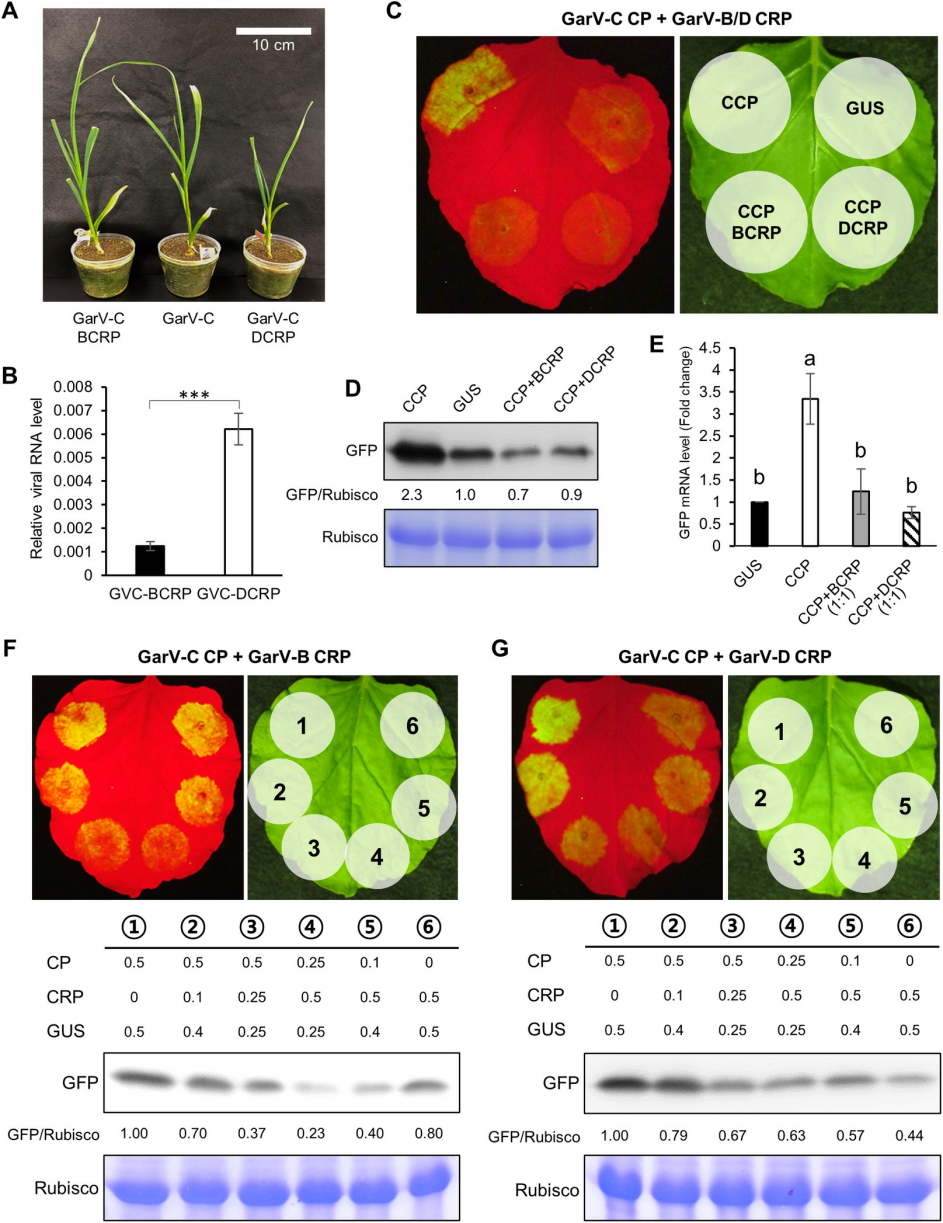
Viral symptoms and the accumulation levels of GarV-C recombinants containing BCRP and DCRP. (A) Plants differed in size by 21 dpi with GarV-C, GarV-C-BCRP or GarV-C-DCRP. (B) The levels of viral RNA accumulation in systemic leaves determined using real-time RT-PCR at 12 dpi. Mean values (±SEM) for CP/actin (*n* = 4) were analyzed for differences using a two-sided Student’s *t*-test (***P < 0.001). (C-D) Antagonistic effect between GarV-C CP (CCP) and CRP-FLAGs of GarV-B and GarV-D. The effect of co-expression of the two proteins on overall RSS activities were examined as described for Fig 7A and 7B. (E) The same tissues were used for real-time RT-PCR to compare the *GFP* mRNA levels as described in Fig 2C (*n* = 3 or 4). (F, G) Antagonistic interaction between GarV-C CP (CCP) and BCRP/DCRP in RSS activity observed when the expression ratio of *CP* to *CRP* was gradually changed. Overall RSS activity was examined in agroinfiltration patches expressing the constructs [GarV-C CP + GarV-B CRP] (F) and [GarV-C CP + GarV-D CRP] (G) at various *CP*/*CRP* expression ratios. The effect of co-expression of the two proteins on overall RSS activities were examined as described for Fig 7F and 7G. The CBB-stained Rubisco large subunit (Rubisco) is shown as a loading control (D, F, G).

Finally, we examined the effect of *CRP* expression on allexivirus pathogenicity. We constructed a garlic-infectious cDNA clone of GarV-C (S15 Fig), then exchanged the CRP of the infectious GarV-C with that of BCRP or DCRP, and agroinfiltrated garlic plants with the recombinant viruses. As shown in Fig 8A, the GarV-C-DCRP-infected garlic plants were slightly stunted and accumulated significantly higher viral RNA levels compared to GarV-C-BCRP-infected garlic plants (Fig 8B). The expression of various *CRP*s from the recombinant GarV-C clone was confirmed by western blot analysis (S15D Fig). Here, we also found that the accumulation levels of DCRP and BCRP-mNES were more abundant than that of BCRP. These results suggest that CRP indeed functions as a pathogenic determinant when produced by an allexivirus.

Because CRP might contribute to viral pathogenicity, we assessed the antagonistic effect of CRP on the RSS activity of GarV-C CP in the presence of either BCRP or DCRP. We found that GarV-C CP had RSS activity, which could be inhibited by either BCRP or DCRP (Figs 2D and 8C–8E); thus trans-inhibition between CP and CRP derived from different garlic viruses indeed occurs. Increasing the levels of both BCRP and DCRP more efficiently repressed the RSS activity of GarV-C CP, and BCRP more strongly repressed the overall RSS activity than DCRP did (Figs 8F, 8G, S13C and S13D). These results suggest that both BCRP and DCRP can negatively regulate allexivirus infection by interfering with the CP’s RSS activity, and BCRP, which is in the cytoplasm, can more strongly reduce CP’s RSS activity and viral pathogenicity than DCRP can.

## Discussion

### Allexiviruses synthesize a short sgRNA for *CRP* expression

Few reports have focused on how viral *CRP* genes are expressed in plant viruses; only one describes *CRP* expression from a sgRNA in PMTV [28]. For allexiviruses, an approximately 1500-nt-long sgRNA was reported, which includes the *CP* and *CRP* ORFs [6].

We previously proposed a possible coupled translation of *CP* and *CRP* via the termination/reinitiation strategy. However, here, our gene expression analysis of *CRP* using the CP-CRP constructs revealed that the translation from the *CP* ORF was not efficiently coupled with *CRP* expression (Fig 1). We thus presumed that another mechanism was required for *CRP* expression. In this study, we demonstrated the presence of possible sgRNAs for *CRP* (sgRNA-CRP) (Figs 3B and S4D) and found that CRPs were generated from the short sgRNA-CRP constructs that mimicked sgRNAs (Fig 3C and 3D) by agroinfiltration, but not when the CP-CRP constructs were used. These results indicate that allexiviruses may synthesize sgRNA-CRPs for *CRP* expression. However, these results do not negate the possibility that some ancestor virus(es) of allexiviruses may have used the termination/reinitiation strategy for *CRP* expression.

Our *CRP* expression analysis using various viral RNA constructs (S2 Fig) demonstrated that the GarV-B sgRNA-CRP had two putative translation initiation sites (or ribosomal binding sites) including (1) the 5′ noncoding region (NCR) for *CRP* and (2) an IRES inside of the *CRP* ORF, whereas the GarV-D sgRNA-CRP contains only one ribosomal binding sites at the 5′ NCR (Fig 3). Considering that the lack of IS in GarV-B enhanced *GFP* expression but not the CRP-GFP expression (Fig 3E and 3F), the ISs may have a critical role in *CRP* gene expression from sgRNA-CRP to compete with the IRES, perhaps in binding to ribosomal RNAs.

### Intracellular localization determines the RSS activity of allexivirus CRPs

Our assay of RSS activity revealed that not all allexivirus CRPs have RSS activity and that the subcellular localization is important for that activity. For example, mutational analyses of the NS3 protein of RSV revealed that the nuclear-localized NS3 functions in suppressing RNA silencing [19,37]. On the other hand, tomato bushy stunt virus (TBSV) P19, which is primarily localized in the cytoplasm, lost its RSS activity when translocated into the nucleus [38]. Cytoplasmic CMV 2b plays a primary role in suppressing host antiviral RNA silencing by binding to small RNAs [39,40]. Recently, our group reported that exportin-mediated nuclear export is important for strong RSS activity of CMV 2b [41]. In the present study, we found that exchanging the sequences corresponding to the NES motif between BCRP and DCRP impaired the cytoplasmic localization and RSS activity of BCRP, while the NES-inserted DCRP was observed in the cytoplasm and acquired RSS activity (Figs 4 and S8). These results indicate that exportin-mediated nuclear export is important for the RSS activity of CRP and that the group of allexiviruses in which CRP does not contain an NES lacks RSS activity. Our prediction of an NES motif in CRPs of allexiviruses revealed that the six allexiviruses consist of two groups; one group contains an NES and the others do not (S6 Fig). Indeed, GarV-X CRP, which was predicted to contain an NES, has RSS activity [9], supporting our hypothesis. Considering that all the CRPs of the six allexiviruses contain an NLS motif (S6 Fig), and thus must be imported into the nucleus, only the CRPs that are exported from the nucleus can suppress RNA silencing.

### Link between autophagy and allexivirus pathogenicity

Our data demonstrated that autophagy counteracted the BCRP-mediated suppression of RNA silencing, whereas DCRP was less affected by autophagy. BCRP interacted with ATG8a and is localized in the cytoplasmic autophagosomes, whereas DCRP did not induce production of autophagosomes (Fig 5A and 5C). In addition, the treatment with the autophagy inhibitor E64d greatly increased the level of BCRP and RSS activity, and the treatment with the autophagy inducer BTH negatively affected BCRP accumulation and RSS activity, whereas both the inhibitor and the inducer had relatively a mild effect on DCRP accumulation (Fig 6). Similarly, *ATG7* silencing resulted in a higher accumulation level of BCRP than that of DCRP (Fig 6C). Because the BCRP-induced autophagosomes were detected in the cytoplasm, the cytoplasmic CRPs seem to be the main target of autophagy. Zhang et al. (2018) previously demonstrated that the NLS-mutated CRP of GarV-X was localized in the cytoplasm as small puncta [9], supporting our hypothesis that cytoplasmic allexivirus CRPs are targeted by autophagy. On the other hand, DCRP, which lacks an NES motif and RSS activity, did not induce autophagosome formation in the cytoplasm or interact with ATG8a, and when the NES was inserted into DCRP, the autophagosomes were clearly detected in the cytoplasm (Figs 4 and S8), indicating that the DCRP escapes autophagic degradation by being in the nucleus. Together, we presume that intracellular distribution of allexivirus CRPs contributes to regulating protein degradation through autophagy.

With regard to the RSS activity of CP in the presence of CRP, we found that the RSS activity of BCP was strongly repressed by BCRP expression, where DCRP had a relatively moderate inhibitory effect on the RSS activity of DCP (Fig 7). Given that the majority of CPs are in the cytoplasm, the cytoplasmic CRPs seems to function as a negative regulator of the RSS activity of CPs. Therefore, in the presence of CP, DCRP without an NES may behave better than BCRP with an NES in maintaining CP’s RSS activity, and thus overall viral RSS activity. Supporting this hypothesis, the RNA level of the recombinant GarV-C expressing DCRP was relatively higher than that of the recombinant expressing BCRP (Fig 8A and 8B). In addition, Cafrune et al. (2006) previously reported that GarV-A, a D-type allexivirus, induces more severe symptoms in garlic plants than the I-type allexivirus GarV-C does (S1 Fig) [3].

By regulating allexivirus RSSs, autophagy seems to play an important role in viral infection; it decreases cytoplasmic CRP accumulation and thus directly represses the RSS activity of CRP, whereas the reduction of CRP in the cytoplasm elevates free CP levels and CP’s RSS activity. We consider that CP rather than CRP has the main role in suppressing host antiviral RNA silencing because GarV-C carrying DCRP has higher virulence than BCRP even though DCRP lacks RSS activity (Figs 2, 8A and 8B). Consequently, we believe that autophagy enhances the RSS activity of CPs by removing CRPs from the cytosol. Allexiviruses may have evolved to exploit the host autophagy system to use CP as the major viral RSS by regulating CRP levels.

### Possible association between allexivirus CP and CRP

We also suspect that a weak interaction detected between CP and CRP may have erased the RSS activity of the two proteins (S14 Fig). In addition, based on the results of the co-IP experiments, the modified CP but not the native one seemed to have bound to CRP. The mode and degree of the CP posttranslational modification are not known at this moment; the difference of about 5 kDa in size to the calculated value suggests such modification as phosphorylation and ubiquitination. According to the 3D structures predicted in AlphaFold2, we here propose some hypothetical CP-CRP interaction models *in silico* (S16 Fig). Despite of the fact that the protein sequences were quite different (62.7% and 55.9% homology for CP and CRP, respectively) between the two viruses, we noticed that the two docking models for the BCP-BCRP and DCP-DCRP associations with the lowest total energy were very similar. In addition, the positively charged areas on CRP seem to contact the negatively charged areas in CP (S16 Fig), and the contact areas (a1) include one or two intermolecular hydrogen bond(s) (H-bond), which would enhance the CP-CRP association. Although we do not have any experimental evidence on the CP post-translational modification at this moment, those *in silico* docking models suggest that the electrostatic status and the H-bond formation may somehow affect the CP-CRP association. We thus consider that without characterizing the possible CP post-translational modification, we could not understand how such a modification affects the CP-CRP interaction. The mechanism by which the RSS activity of CP and CRP disappears upon co-expression of the two proteins would be given based on the results of those experiments in the future.

### CRP may be the key driving force for allexivirus evolution

In the phylogenetic trees, I-type and D-type CRPs were clearly diversified with strong phylogenetic supports suggesting that ISs between the *CP* and *CRP* genes contributed to the evolution of both genes and thus of allexiviruses. When trees were created using the CP-CRP sequences including the ISs, I-type allexiviruses formed a monophyletic clade from the D-type, indicating that I-type viruses may have evolved from the D-type (S1A Fig). Here, because the *CP* gene and the *CRP* gene of the two outgroup viruses (citrus yellow vein clearing virus [CYVCV] and Indian citrus ringspot virus [ICRV]) overlap, the virus did not belong to the I- or the D-type. The topology of the tree constructed for the CRPs was similar to that of the CP-CRP tree (S1C and S1E Fig). However, the topology of the tree based on the CPs was different from that based on the CRPs (S1B and S1D Fig); the CP trees based on amino acid and nucleotide sequences indicated that I-type viruses were not generated from the monophyletic clade and that I-type GarV-C was placed as a sister clade of the D-type viruses. These results thus suggest that the *CP* and *CRP* genes evolved independently, that the CP of the D-type viruses might have evolved from the CP of I-type, and that GarV-C is a possible ancestor of the D-type. The discrepancy in the phylogenetic topology between the CPs and the CRPs may be explained by considering different levels of selection pressures on the two genes. The CRP of ShVX seems to be an intermediate between the I-type and D-type allexiviruses. While the CRPs between the I- and the D-type seem to be very different in gene expression strategy and nucleocytoplasmic localization, the CPs seem to have a similar level of RSS activity regardless of their I-type or D-type aside from their original function of forming particles.

**Fig 9:**
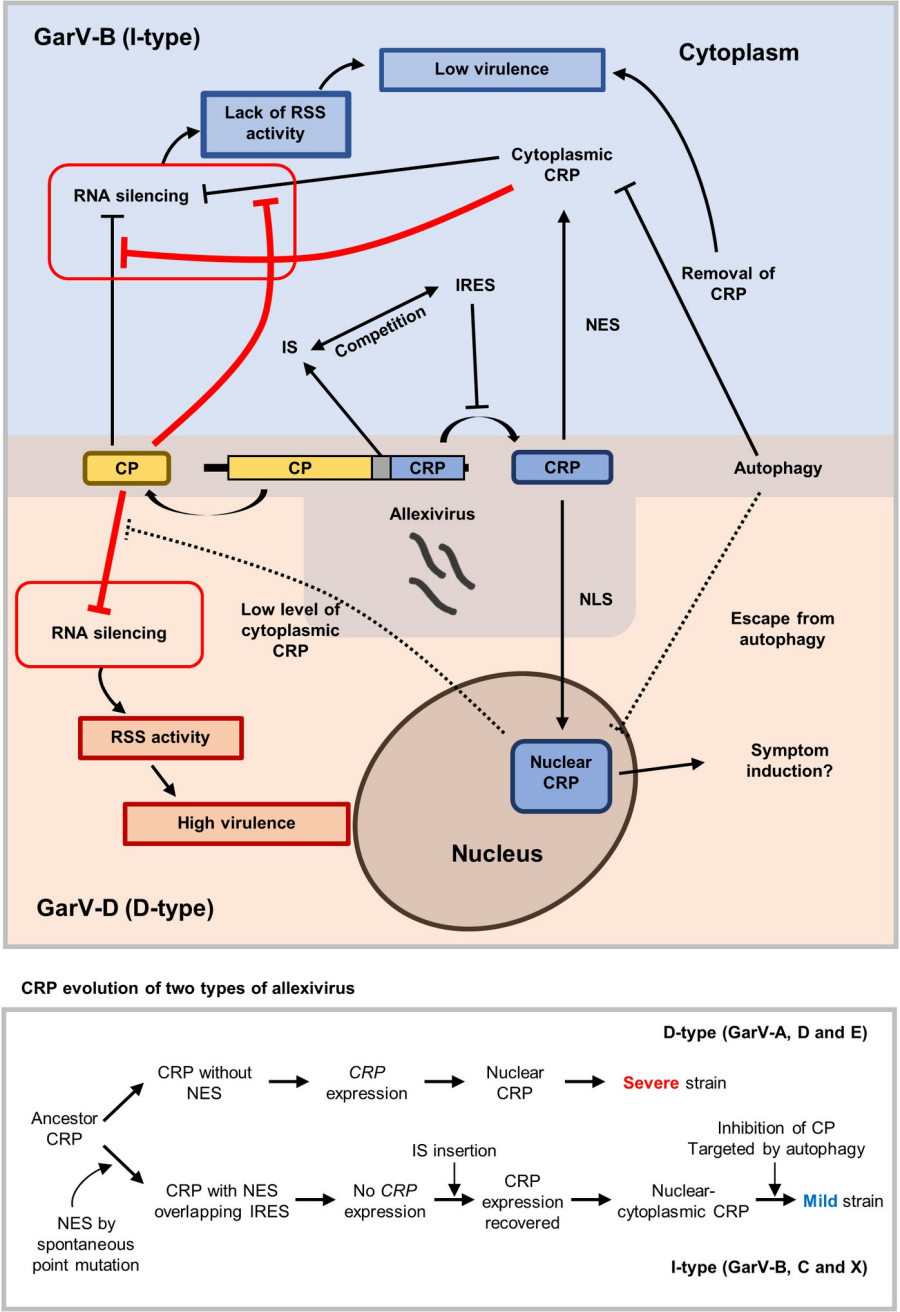
Model of the functions of allexivirus CP and CRP against RNA silencing and autophagy. CRPs of I-type allexiviruses (GarV-B, C and X) contain an NES for their transport from nucleus to cytosol, and cytoplasmic CRPs function in the suppression of antiviral RNA silencing. The presence of an IRES in I-type allexivirus *CRP*s reduces CRP translation. To recover *CRP* expression, I-type allexiviruses must have acquired the inserted sequences (ISs) between the *CP* and *CRP* genes, and the ISs can compete with IRES for ribosome-binding in the CRP ORF for CRP translation. However, the accumulation of cytoplasmic CRP tends to inhibit the RSS activity of CP while the RSS activity of CRP is also impaired by CP. In addition, as a counter-counterdefense against the CRP-mediated suppression of RNA silencing, plants may use autophagy to degrade cytoplasmic CRPs; thus, I-type allexiviruses would have relatively low virulence because host RNA silencing will be dominant over viral RSSs. Therefore, to avoid host autophagy, a different type of allexivirus (GarV-A, D and E) may have developed CRPs that do not have an NES motif and are localized in the nucleus. Such allexiviruses may have evolved to lose ISs (Deletion [D]-type), adjusting the *CRP* expression level, which may interfere with the RSS activity of CP before the synthesized CRPs are translocated into nucleus. CRPs of D-type allexiviruses do not interfere with the RSS activity of CP and are not targeted by autophagy because they are in the nucleus; thus, they would have relatively high virulence. Consequently, the ancestor allexiviruses may have evolved two scenarios for survival, and the scenario in play might be determined by the environment, including the host plants and geographic factors.

Although we consider that our hypothesis on the evolution of allexiviruses should be further confirmed by increasing the number of allexivirus CP and CRP sequences in the phylogenetic analyses, we here propose a working model for the plausible functions of the CP and CRP in viral infection and the possible evolutionary scenarios (Figs 9 and S17). The IRES inside of the GarV-B *CRP*, which overlaps with the NES motif, seems to be important for the translocation of the I-type from the nucleus to the cytoplasm where it can again serve as an RSS. However, the cytoplasmic CRP will be targeted by host autophagy because ATG8a can bind to GarV-B CRP; CRPs may be degraded via autophagy, resulting in generation of a mild strain (Fig 9). The presence of IRES negatively affects GarV-B *CRP* expression by competing with the CRP 5′ NCR in binding to ribosomal RNAs for translation. To restore and enhance *CRP* expression, the CRPs that have an NES (GarV-B, C and X) must have evolved to integrate ISs just before the *CRP* ORF. An increase in the cytoplasmic GarV-B CRP level reduces the RSS activity of the CP and thus virulence of the virus. On the other hand, the D-type *CRP* genes lack IS, IRES, NES and RSS activity. Instead, they are not significantly degraded by host autophagy because they are localized in the nucleus. The CP can thus fully serve as a strong RSS, making the virus a severe strain.

In this study, we demonstrated that allexiviruses had two evolutionary scenarios, one for the I-type and another for the D-type, which are both well-crafted but mutually exclusive. Which scenario is involved will depend on the selection pressure(s) that the allexivirus encounters.

## Materials and methods

### Plant materials

Garlic bulbs were purchased at the market. *N*. *benthamiana* seedlings were grown in pots with 16 h light/8 h dark at 24°C in a growth chamber for later use for agroinfiltration and the PVX inoculation experiments. Onion bulbs were also purchased at the market. Virus-free garlic bulbs were obtained from the Ornamental Plants and Vegetables Research Center of Hokkaido.

### *Agrobacterium*-mediated transient expression

The CP-CRP sequences of GarV-B (GenBank LC731731) and GarV-D (GenBank LC731732) were inserted into the pBE2113 binary plasmid vector using the XbaI and the SacI restriction enzymes. *Agrobacterium* was transformed with the recombinant constructs by the conventional freeze–thaw method, and the transformants were cultured in yeast extract and peptone (YEP) medium (1% yeast extract, 1% peptone and 0.5% NaCl, all w/v). The bacterial cells were harvested by centrifugation (8,000 rpm, 2 min), and then resuspended in resuspension buffer (10 mM MgCl_2_, 10 mM MES and 0.2 mM acetosyringone). After 2 h at 25°C, *N*. *benthamiana* leaves were infiltrated with the bacterial suspensions using a 1-mL syringe without a needle.

### Construction of recombinant PVX and GarV-C clones containing allexivirus CP-CRP or CRP

The CP-CRP and CRP sequences of GarV-B and GarV-D were amplified from garlic total RNA by RT-PCR using the primers in S1 Table. The PCR products were cloned into the PVX vector between the SalI/ClaI or SalI/XhoI restriction enzyme sites to create recombinant PVXs harboring either CP-CRP or CRP sequence. The CRPs were tagged with FLAG for western blot analysis. The recombinant PVX constructs were then transcribed *in vitro* using T7 RNA polymerase (Takara) to obtain PVX transcripts to use as inocula. Four-week-old potted plants of *N*. *benthamiana* were mechanically inoculated with the PVX transcripts in 0.1 M phosphate buffer. For the construction of a GarV-C infectious clone, the complete cDNA sequence of GarV-C genomic RNA (GenBank: AB010302.1) was synthesized and inserted into the pBI101 binary vector under the control of the 35S promoter (pBI-GarV-C) (S15 Fig). The *CRP* ORF in pBI-GarV-C was exchanged with either the BCRP ORF or DCRP ORF using the integrated cloning site (StuI and SpeI restriction enzyme sites) creating the constructs pBI-GarV-C-BCRP and pBI-GarV-C-DCRP. Garlic shoots were agroinfiltrated with *Agrobacterium* cells containing an infectious clone as described above.

### Observation of GFP fluorescence

*Agrobacterium* was transformed with the binary vector pBE2113 harboring the constructs DsRed-NbATG8a, CRP-GFPs or its putative sgRNA-CRP-GFP (S2 Fig), and then *N*. *benthamiana* leaves were infiltrated with the bacterial suspensions (OD_600_: 1.0). At 2 dpa, infiltrated patches were observed for GFP florescence using an epifluorescence microscope (Leica DMI 6000B).

### Western blot assay

For detecting CP or CRP-FLAG expressed through the PVX vector, the leaf tissues were excised from infected plants, and the total protein extracts were separated in a 14% polyacrylamide gel using SDS-PAGE. CP and CRP-FLAG were detected using western blots with anti-CP antibodies and anti-FLAG monoclonal antibody, respectively. To detect CRP-GFPs extracted from agroinfiltrated *N*. *benthamiana* leaves, anti-GFP antibodies were used as described before [41].

### RNA silencing suppression assay

For examining RSS activity of CPs and CRPs in *N*. *benthamiana* and onion plants, *GFP*, *CP* and CRP-FLAG genes were cloned into the pBE2113 binary vector, and *Agrobacterium* was transformed with those constructs. Bacterial cultures were adjusted to OD_600_ of 1.0 for *N*. *benthamiana* and 0.2 for onion in the resuspension buffer described above. The bacterial suspensions of GFP and CP/CRP were mixed at 1:2 with the resuspension buffer for *N*. *benthamiana* and 1:1 for onion. Plants were agroinfiltrated as described above, and GFP fluorescence was assessed using blue light at 5 dpa. GFP was detected in western blots using anti-GFP antibodies and quantified using Multi Gauge Software (BAS-1000, Fujifilm, Tokyo, Japan). For the assay using onion cells, 0.2~0.3 mL of the bacterial inocula were used to infiltrate onion bulbs with a 1-mL syringe and 0.5 × 25 mm needle. Cells were checked for GFP fluorescence at 3 dpa using an epifluorescence microscope. The fluorescence intensity was quantified using LAS AF software (Leica Microsystems).

### Real-time RT-PCR

Total RNA from leaves of infected *N*. *benthamiana* plants was treated with DNase I, then cDNAs were synthesized using the PrimeScript RT reagent kit (Takara). The primers used for real-time RT-PCR are listed in S1 Table. For quantification of PVX RNA levels, real-time RT-PCR was performed using Powerup SYBR Green Master Mix (Applied Biosystems) with PVX-CP5-110/PVX-CP3-110 primer pair. To analyze the *GFP* mRNA levels, S65T-5-168/S65T-3-168 primer pair was used. The 60S ribosomal protein L23 gene (*RPL23*) was co-amplified with Nb-L23-5-110/Nb-L23-3-110 primer pair as a reference. For quantifying GarV-C RNA levels in the inoculated garlic plants, primer pair Alle-CP3-750/Alle-CP5-750 was used. Partial *Actin* sequence was amplified as a reference using primer pair Garlic-act-rt-F2/Garlic-act-rt-R2.

### Northern blot analysis for sRNA detection

After high-molecular weight RNAs in total RNA were first precipitated by 20% polyethylene glycol #6000, low-molecular weight (LMW) RNAs were recovered from the supernatant by ethanol precipitation. The LMW RNAs were then separated by electrophoresis in a 15% polyacrylamide gel and transferred onto the Amersham Hybond-N^+^ membrane (GE Healthcare). Northern blot hybridization was performed essentially according to the method by Goto *et al*. (2003) [42]. An antisense *GFP in vitro* transcript labeled with digoxigenin (DIG) was used as a probe to detect *GFP*-derived sRNAs. The signals were detected using anti-digoxigenin-AP Fab fragments and CDP-Star (Roche).

### 5′ RACE to detect the possible 5´ ends of sgRNAs for CRP

The manufacturer’s protocol for the kit, 5´-Full RACE Core Set (Takara, Shiga, Japan) was used with some modifications (S3 Fig). The 3′ ends of the double-stranded cDNAs were polyadenylated by recombinant terminal transferase to identify the exact 5′ ends for sgRNAs. RT-PCRs were then performed using an oligo dT primer and a virus-specific primer (S1 Table) that hybridizes to the sequence in the *CRP* ORF. The nucleotide sequences of the 5′ RACE products were determined. For the isolation of sgRNAs encapsidated in the particles, immunoprecipitation was conducted using anti-[GarV-D+GarV-X] antibodies and Dynabeads Protein G Immunoprecipitation Kit (Invitrogen) following the manufacturer’s instruction. Viral RNA was extracted from the purified particles by phenol/chloroform.

### Bimolecular fluorescence complementation (BiFC) assay

The genes of interest were fused to the N- and C-terminal of mVenus (VN and VC) fluorescent protein and cloned into the pBE2113 binary plasmid. *N*. *benthamiana* leaves were agroinfiltrated as explained above. The expression of the VN- and VC-fused proteins were confirmed by western blot analysis (S7 Fig) using anti-GFP antibodies (MBL). Cells were checked for mVenus fluorescence using epifluorescence microscopy (Leica DMI 6000B) with filter cube L5 (480/40 nm excitation, 505 nm dichroic mirror, 527/30 nm barrier) at 2 dpa.

### Chemical treatments

LMB (40 nM) was applied to leaves at 2 dpa using the method of Kim et al. (2022) [41]. Leaves were treated with E64d using minor modifications of the method of Jeon et al. (2017) [43]. E64d (20 μM) in PBS buffer containing 2% DMSO was used to infiltrate the agroinfiltration patches at 24 h post agroinfiltration (hpa). For the BTH treatment, BTH in distilled water (1 mM) was sprayed on the agroinfiltrated leaves at 24 hpa as described by Kobayashi et al. (2020) [44]. The leaf tissues treated with E64d or BTH were excised after 16 h for western blotting or after 4 days for quantifying RSS activity.

### VIGS of *N*. *benthamiana ATG7* by the CMV vector

The CMV-A1 vector (A1) was used to silence *ATG7* by VIGS [45]. A partial *ATG7* sequence (190-nt) was amplified by RT-PCR from *N*. *benthamiana* total RNA using ATG7-5-190-Ml/ATG7-3-190-St primer pair (S1 Table). We selected the *ATG7* gene (Genbank KX369398.1) as the target of VIGS because it had been previously proven that silencing of this gene was sufficient to inhibit autophagy in *N*. *benthamiana* [36]. The *ATG7* PCR fragment was cloned into the A1 vector in antisense orientation to generate A1-ATG7. CMV RNA1, RNA3 and the recombinant A1 were *in vitro* transcribed using T7 RNA polymerase (Takara), and those transcripts were inoculated to *N*. *benthamiana* plants. CMV infection and *ATG7* silencing were confirmed by real-time RT-PCR using primer pairs listed in S1 Table.

### Phylogenetic analysis

The CP-CRP sequences for the GarV-B (Spain, GenBank LC731731) and GarV-D (China, GenBank LC731732) isolates that were determined in this study and for GarV-A, GarV-B, GarV-C, GarV-D, GarV-E, GarV-X, and ShVX retrieved from GenBank were used in a phylogenetic analysis. Two species of genus *Mandarivirus*, CYVCV (GenBank MF563877) and ICRV (GenBank NC_003093), were used as outgroups because they had the highest homology with allexiviruses among the viral genomes in the GenBank database. Multiple sequence alignment was created using MUSCLE v3.8.31 [46] with default settings. The phylogenetic tree based on CP-CRP nucleotide sequences was inferred using the Bayesian method implemented in MrBayes v3.2.7 [47]. In addition, we also created phylogenetic trees based on the nucleotide and amino acid sequences of the *CP* and *CRP* genes. The GTR model with gamma distribution, among-site rate variation and a proportion of invariable sites was applied for the nucleotide sequences, and the JTT model was applied for the amino acid sequences. Markov chain Monte Carlo sampling was performed for 3,000,000 iterations with sampling every 3,000 steps. The first 25% of the samples were discarded as burn-in state. The tree was visualized using R package ggtree [48].

### Accession numbers

All the sequencing data used in this study can be found in the GenBank database. The newly generated sequencing data of GarV-B CP-CRP (GenBank LC731731) and GarV-D CP-CRP (GenBank LC731732) are available in the GenBank database.

## Supporting information

S1 Fig Phylogenetic analyses of allexiviruses based on the CP and CRP sequences. (A) Phylogenetic tree constructed based on nucleotide sequences of the CP-CRP region. The phylogenetic tree (left side) was constructed using the Bayesian method as explained in the Result section. Internal node values indicate posterior probabilities (only ≥50% are shown). CYVCV (MF563877) and ICRV (NC_003093) were used as the outgroups. Red text (Spain and China) indicates sequences obtained in this study; black text indicates GenBank accessions. The multiple sequence alignments around the insert sequences are identified on the right. Phylogenetic trees based on (B) *CP* nucleotide sequences, (C) *CRP* nucleotide sequences, (D) CP amino acid sequences and (E) CRP amino acid sequences. The trees were constructed using the Bayesian method as explained in the Method section. Internal node values indicate posterior probability (only ≥50% are shown). CYVCV (MF563877) and ICRV (NC_003093) were used as the outgroups. Red font isolates (Spain and China) are the sequences obtained in this study while black font isolates are the GenBank accession number of each isolate.
(PDF)

S2 Fig Diagrams of GarV-B and GarV-D constructs for gene expression after agroinfiltration. GarV-B and GarV-D sequences were cloned into the plant expression vector pBE2113.
(PDF)

S3 Fig Schematic flow of 5′ RACE to detect the 5’ ends of possible sgRNAs. Detailed explanation of the 5′ RACE is given in the Method section.
(PDF)

S4 Fig Detection of sgRNA-CRP encapsidated in allexivirus particles. (A) Flowchart for detection of encapsidated sgRNA-CRP. The crude extract of allexivirus-infected garlic tissue was co-incubated with antibody-bound protein G dynabeads (Invitrogen), then the virus-bound beads were collected using a magnet. After elution of the viral particles from the beads, RNA was extracted. The 5′ end sequences of the extracted sgRNA-CRPs were analyzed by 5′ RACE as explained in S3 Fig. (B) Validation of specific precipitation of viral particles. Semi-quantitative RT-PCRs of CP-CRP regions using RNA from the immunoprecipitated sample (IP) and primer pair of Alle-CP5-750 and Alle-CP3-750 were conducted. Antibodies were not added to the negative control (NC). The number of cycles is given at the top of the gel. Size marker (M): 1 kbp DNA marker. (C) Agarose gel of 5′ RACE PCR amplicons from GarV-D and GarV-X sgRNAs in the purified viral particles. Some discrete bands, which may have been generated from sgRNA-CRPs, were observed (arrowheads). Old and New Ex-Taqs are TaKaRa Ex Taq and PerfectShot Ex Taq (Takara), respectively. Size marker (M): 50 bp DNA marker. (D) Map of 5′ end sequences of possible sgRNA-CRPs isolated from GarV-D and GarV-X particles. Red and blue arrows indicate the 5′ end nucleotide of the PCR products of GarV-D and GarV-X, respectively. Number above the arrow indicates the number of ends detected.
(PDF)

S5 Fig Alignment of nucleotide sequences of GarV-B and GarV-D CRPs. The positions of IRES and translation initiation site in GarV-B CRP are indicated.
(PDF)

S6 Fig Prediction of an NES motif in allexivirus CRPs. (A) Map of predicted nuclear localization signal (NLS) and nuclear export signal (NES) in the CRP amino acid sequences. (B) Prediction of NES motifs in CRPs of six allexiviruses by the program NetNES 1.1. Here, residues with NES scores higher than the threshold (red line) were predicted as putative NESs.
(PDF)

S7 Fig Western blot analyses to verify the expression of CRPs, XPOI and ATG8a in the experiments in Figs 4 and 5 for the microscopic observation, BiFC and suppression of GFP silencing. GFP- or mVenus (VN or VC)-fused proteins were detected using anti-GFP (anti-mVenus) polyclonal antibodies. The FLAG-tagged CRPs were detected using an anti-FLAG antibody. LMB, leptomycin B.
(PDF)

S8 Fig RSS activity of DCRP with a NES. (A) Effect of the NES insertion in DCRP on the DCRP intracellular localization. The sequenceregion of DCRP corresponding to NES was replaced with that of BCRP (S6A Fig) to construct DCRP-NES. GFP-fused DCRP and DCRP-NES were expressed in *N*. *benthamiana* via agroinfiltration. Subcellular localization of the GFP fusion proteins were observed. Scale bars: 50 μm. (B) RSS activity of DCRP-NES. The RSS activities of GUS, FLAG-tagged DCRP and DCRP-NES were compared as described in the Fig 2 legend. (C) The GFP accumulation levels in the agroinfiltrated tissues were analyzed by western blot analysis. The expression of GFP-tagged or FLAG-tagged DCRP mutants were confirmed by western blot analysis as shown in S7 Fig.
(PDF)

S9 Fig Lack of puncta representing autophagosomes during co-expression of CP and ATG8a. CP and DsRed-ATG8a were co-expressed in *N*. *benthamiana* leaves via agroinfiltration. DsRed fluorescence was observed as described in the Fig 5 legend. The two images on the right are close-ups of the white frame in the leftmost image. Scale bars: 50 μm (white), 10 μm (yellow).
(PDF)

S10 Fig Effect of E64d on the BCRP-mNES accumulation. E64d (20 μM) was applied to the leaf tissues where *CRP*s had been expressed as described in Fig 6. The levels of the CRP-GFP accumulation were determined by western blot analysis at 16 h after the E64d treatment.
(PDF)

S11 Fig Silencing of *ATG7* using the CMV VIGS system. (A) Flowchart of *ATG7* silencing in *N*. *benthamiana*. A partial *ATG7* sequence (190-nt) was inserted into the A1 vector (A1-ATG7), and the *in vitro* transcripts of CMV RNA1, RNA3 and the recombinant A1 were used to inoculate to *N*. *benthamiana* plants. At 14 dpi, the CRP-GFP genes were expressed in systemically infected upper leaves by agroinfiltration, and the accumulation levels of CRP-GFP were estimated at 3 dpa by western blot analysis. (B) Symptoms of A1- and A1-ATG7-inoculated *N*. *benthamiana* plants. The pictures were taken at 11 dpi. (C, D) Comparison of the levels of *ATG7* mRNA (C) and CMV RNA (D) between A1-infected and A1-ATG7-infected *N*. *benthamiana* plants. Real-time RT-PCRs were conducted at 14 dpi to measure the *ATG7* mRNA and CMV RNA accumulation levels (*n* = 4). Two-sided Student’s t-test was performed for significant differences (**P < 0.01, ns: not significant).
(PDF)

S12 Fig Subcellular localization of CRPs in the presence of CP. BCRP-GFP and DCRP-GFP were expressed with or without CP by agroinfiltration. The intracellular localization of CRP was observed using Leica DMI 6000B. Scale bars: 50 μm.
(PDF)

S13 Fig Protein accumulation levels of CP and CRP in the agroinfiltrated patches in Figs 7F, 7G, 8F and 8G. As indicated in the legends of Figs 7 and 8, CP and CRP-FLAG were co-expressed in *N*. *benthamiana* leaves by agroinfiltration using the bacterial suspension containing either [BCP+BCRP] (A), [DCP+DCRP] (B), [CCP+BCRP] (C) or [CCP+DCRP] (D). The amount of each construct in the infiltrated *Agrobacterium* suspension is shown at the top of each blot as OD_600_ values of the bacteria (1.0 for total). Western blot analyses were conducted to detect CP and CRP-FLAG using anti-CP antibodies and an anti-FLAG antibody, respectively.
(PDF)

S14 Fig Co-immunoprecipitation (co-IP) to analyze an *in vivo* interaction between CP and CRP. The CP/CRP-GFP complexes were first immunoprecipitated using anti-GFP antibodies, and then CP in the precipitant was detected by western blot analyses using anti-CP antibodies. The red arrowhead indicates the CP in the precipitant. The asterisk represents the light chain of anti-GFP antibodies. The CPs and CRP-GFPs in the total protein extracts, which were used for the IP experiments, were shown in the Input image. CBB-stained Rubisco large subunit is shown as a loading control.
(PDF)

S15 Fig Construction of GarV-C infectious clone. (A) Diagram of the GarV-C infectious clone containing either BCRP or DCRP. The binary vector pBI101 containing the 35S:GarV-C construct (pBI-GarV-C) was created, then the CRP of GarV-C was exchanged with either BCRP or DCRP. (B) Inoculation method of the GarV-C infectious clone. After shoots appeared from potted cloves of a virus-free garlic bulb, the leaves of each plant were infiltrated with *Agrobacterium* containing pBI-GarV-C using a 1-mL needleless syringe. Viral RNA in systemic leaves were quantified by real-time RT-PCR at 12 dpi. (C) Western blot for CP detection to confirm garlic infection by the recombinant GarV-Cs. The arrowhead indicates the CP size. The band just below the CP band seems to be also specific; it may be a processed CP. Leaves infiltrated with the pBI-GarV-C recombinant clones were harvested for western blot analysis at 7 dpa. The GarV-C CP was detected using anti-CP antibodies. Rubisco is shown as a loading control. (D) Verification of the *CRP* expression in the recombinant GarV-C-infected garlic plants at 7 dpa. The CRP region in pBI-GarV-C was replaced by either FLAG-tagged BCRP, BCRP-mNES or DCRP. The recombinant GarV-C constructs were then inoculated into garlic plants by agroinfiltration. The FLAG-tagged CRPs were detected by western blot analysis using an anti-FLAG antibody. Rubisco small subunit (Rubisco) is shown as a loading control. Note that the band for BCRP-mNES was stronger than that for BCRP, suggesting that the presence of a NES in CRP can affect CRP accumulation.
(PDF)

S16 Fig Hypothetical models of the CP-CRP association *in silico*. The predicted tertiary structures of BCP, DCP, BCRP and DCRP were constructed using AlphaFold v2.2.2 [49]. The N-terminal region of CP, which is variable among allexiviruses, was predicted not to form any secondary/tertiary structures regarded as an intrinsically disordered region. The *in silico* docking models for the CP-CRP association were constructed based on the Fast Fourier Transform (FFT) method in ICM-Pro v3.9 (Molsoft, San Diego, CA, USA), and the models with the lowest free energy were selected as the most stable associations. Note that the individual running of FFT docking for BCP-BCRP and DCP-DCRP resulted in very similar interaction models. The electrostatic surface and the contact areas were calculated and visualized in the ICM-Pro. The red- and black-dotted circles (a1 and a2) indicate the contact areas between CP and CRP. Close-up images of the a1 regions are shown to indicate the intermolecular hydrogen bonds (H).
(PDF)

S17 Fig Hypothetical summary of the subcellular localization and function of two allexivirus CRPs. *CP* and *CRP* of GarV-B and GarV-D are expressed from sgRNAs. To avoid the effect of an IRES inside of the *CRP* ORF on *CRP* expression, GarV-B sgRNA-CRP may have acquired an IS between the *CP* and *CRP* ORFs to express *CRP*, while GarV-D sgRNA-CRP, which does not have an IRES, lacks IS. The CPs of the two allexiviruses can suppress RNA silencing in the cytoplasm. Although the CRPs can also function as RSSs in the cytoplasm, they are transported into the nucleus/nucleolus after translation in an importin (IMP)-dependent manner. GarV-B CRPs are then exported from the nucleus by interacting with XPOI; GarV-D CRPs, which lack an NES, are confined to the nucleus. The cytoplasmic GarV-B CRPs can function as an RSS, but because the CRPs and CPs interfere with each other, the two proteins may fail to suppress RNA silencing. Simultaneously, the cytoplasmic GarV-B CRPs are captured by autophagosome receptors and degraded via autophagy. On the other hand, GarV-D CRP may not able to efficiently suppress RNA silencing due to its low levels in the cytoplasm. However, in the nucleus, it escapes autophagy. In addition, because it is localized in the nucleus, the GarV-D CRP cannot interfere with the RSS activity of the CP. GarV-D CP can efficiently suppress RNA silencing.
(PDF)

S1 Table Primers used in this study. (PDF)
