# Supplementary figures and images for: Two mutually exclusive evolutionary scenarios for allexiviruses that overcome host RNA silencing and autophagy by regulating viral CRP expression
Source: PLoS Pathog. 2023 Jun 28;19(6):e1011457. doi: 10.1371/journal.ppat.1011457 (PMC10335701; doi:10.1371/journal.ppat.1011457)

# S1 Fig

**A**

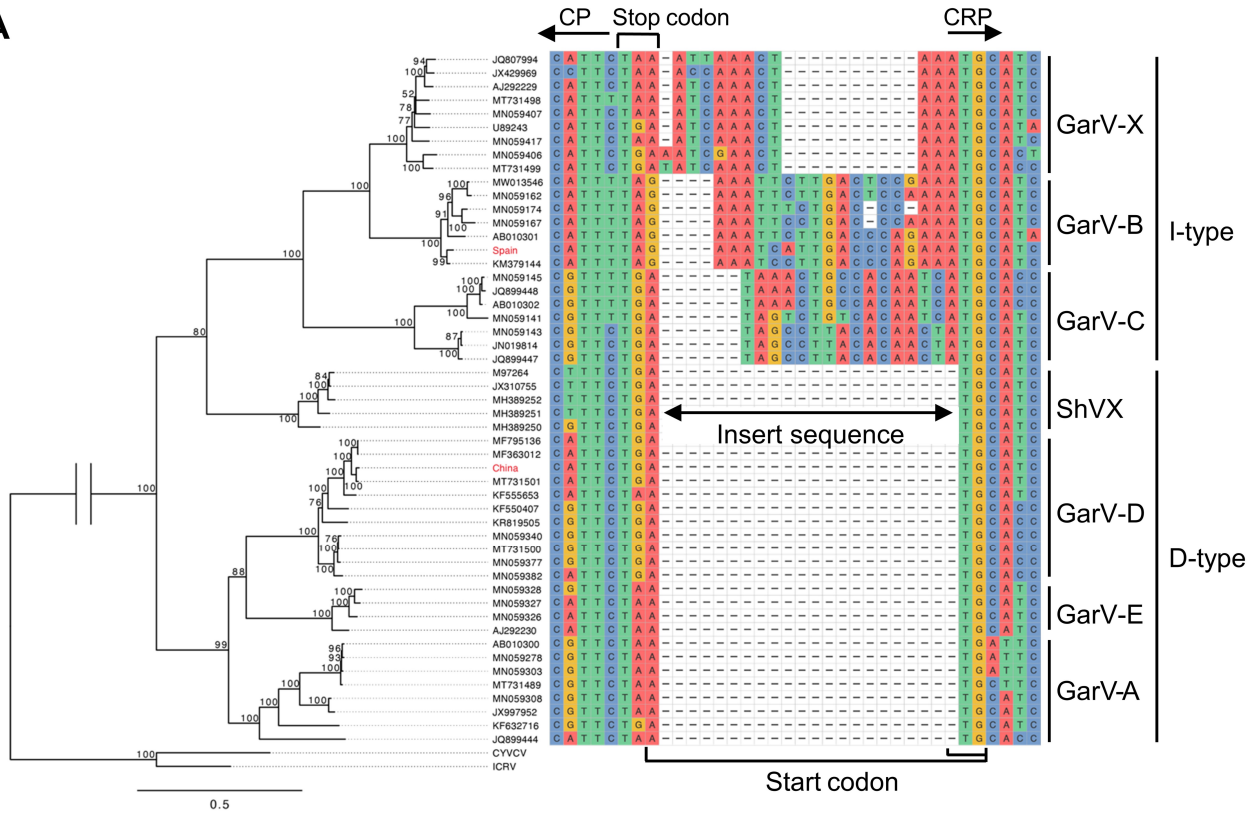

**B**

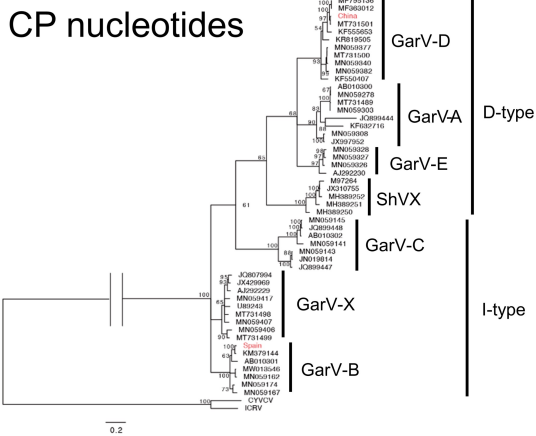

**C**

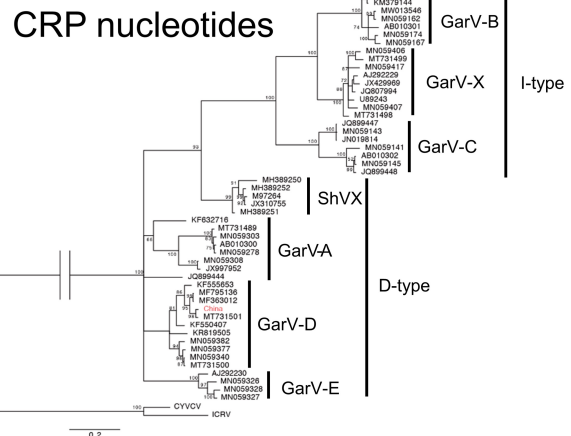

**D**

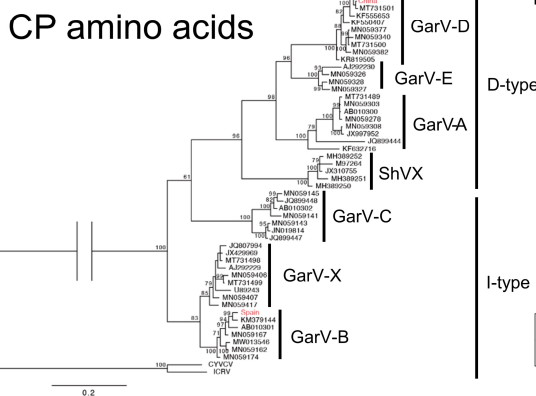

**E**

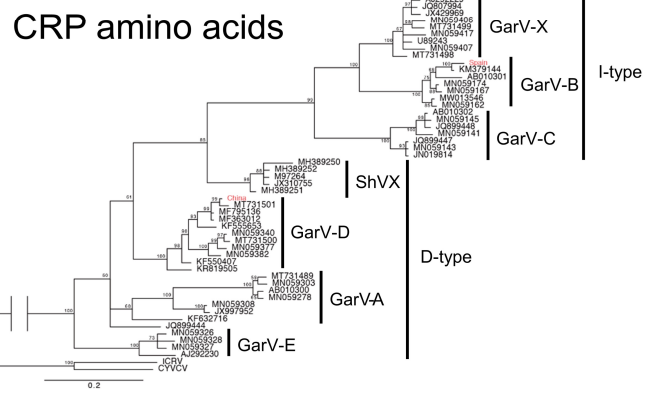

Supplement: S1 Fig — (A) Phylogenetic tree constructed based on nucleotide sequences of the CP-CRP region. The phylogenetic tree (left side) was constructed using the Bayesian method as explained in the Result section. Internal node values indicate posterior probabilities (only ≥50% are shown). CYVCV (MF563877) and ICRV (NC_003093) were used as the outgroups. Red text (Spain and China) indicates sequences obtained in this study; black text indicates GenBank accessions. The multiple sequence alignments around the insert sequences are identified on the right. Phylogenetic trees based on (B) CP nucleotide sequences, (C) CRP nucleotide sequences, (D) CP amino acid sequences and (E) CRP amino acid sequences. The trees were constructed using the Bayesian method as explained in the Method section. Internal node values indicate posterior probability (only ≥50% are shown). CYVCV (MF563877) and ICRV (NC_003093) were used as the outgroups. Red font isolates (Spain and China) are the sequences obtained in this study while black font isolates are the GenBank accession number of each isolate. (PDF) [file ppat.1011457.s001.pdf]

# S3 Fig

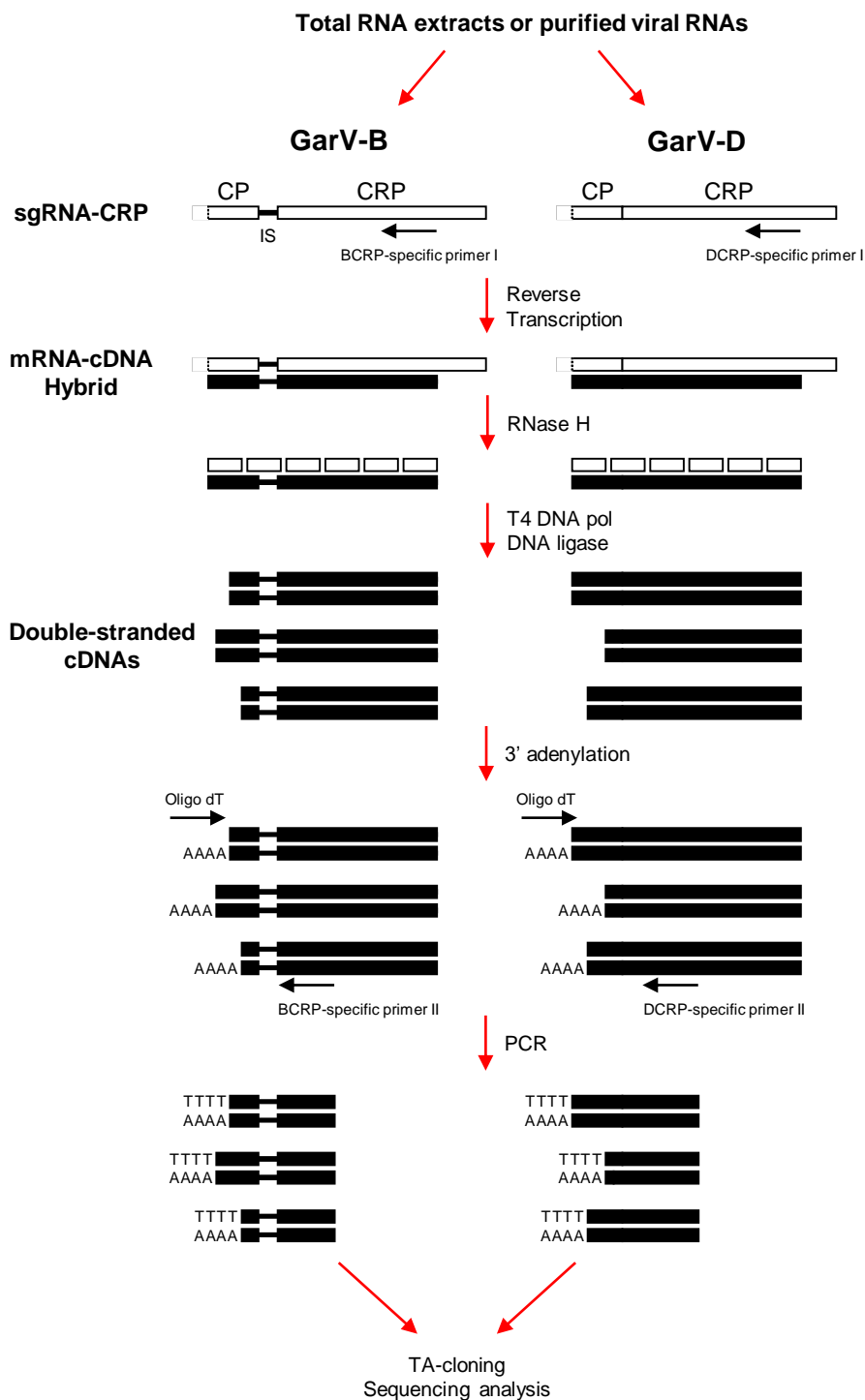

Supplement: S3 Fig — Detailed explanation of the 5′ RACE is given in the Method section. (PDF) [file ppat.1011457.s003.pdf]

S4 Fig

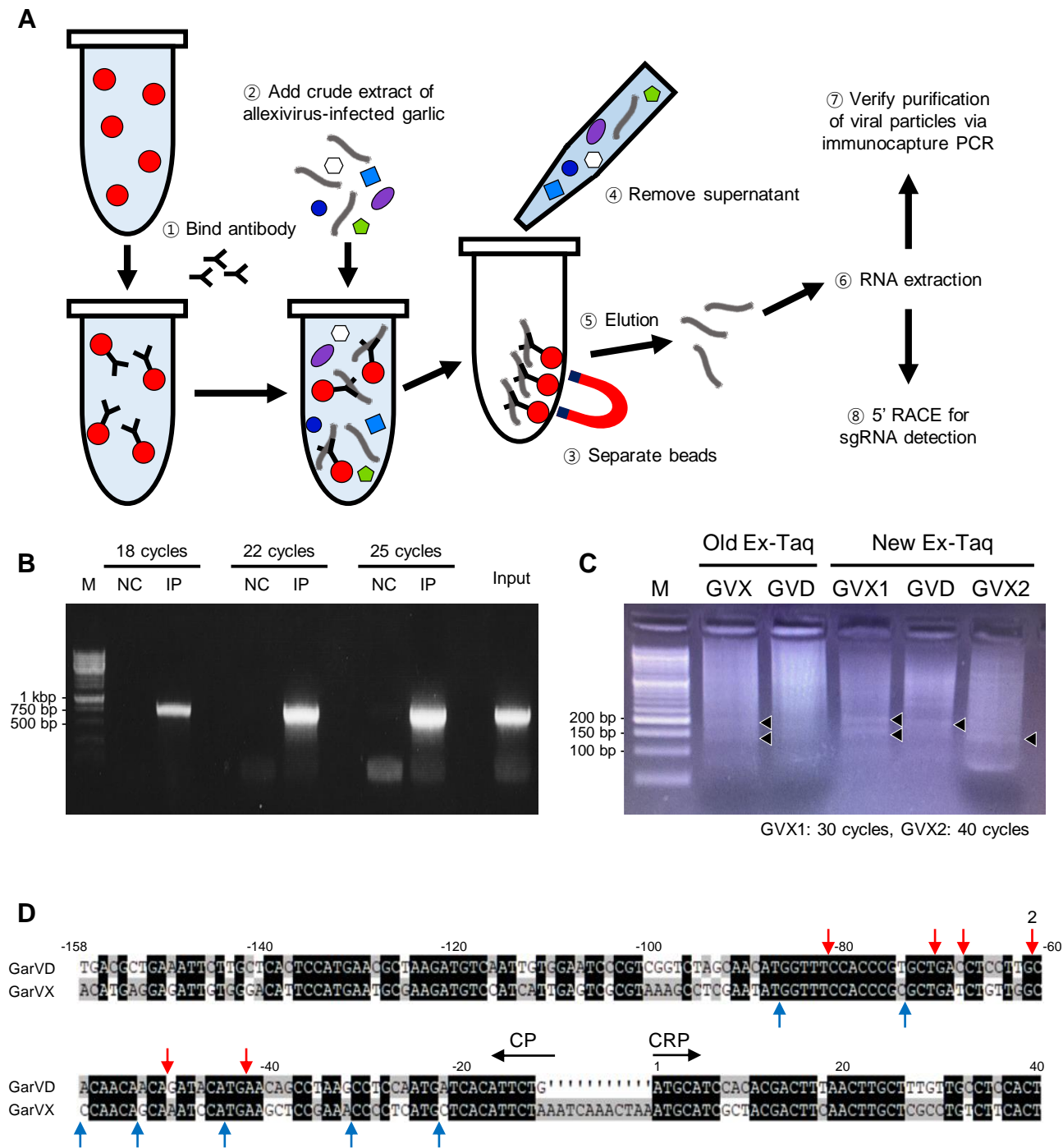

Supplement: S4 Fig — (A) Flowchart for detection of encapsidated sgRNA-CRP. The crude extract of allexivirus-infected garlic tissue was co-incubated with antibody-bound protein G dynabeads (Invitrogen), then the virus-bound beads were collected using a magnet. After elution of the viral particles from the beads, RNA was extracted. The 5′ end sequences of the extracted sgRNA-CRPs were analyzed by 5′ RACE as explained in S3 Fig. (B) Validation of specific precipitation of viral particles. Semi-quantitative RT-PCRs of CP-CRP regions using RNA from the immunoprecipitated sample (IP) and primer pair of Alle-CP5-750 and Alle-CP3-750 were conducted. Antibodies were not added to the negative control (NC). The number of cycles is given at the top of the gel. Size marker (M): 1 kbp DNA marker. (C) Agarose gel of 5′ RACE PCR amplicons from GarV-D and GarV-X sgRNAs in the purified viral particles. Some discrete bands, which may have been generated from sgRNA-CRPs, were observed (arrowheads). Old and New Ex-Taqs are TaKaRa Ex Taq and PerfectShot Ex Taq (Takara), respectively. Size marker (M): 50 bp DNA marker. (D) Map of 5′ end sequences of possible sgRNA-CRPs isolated from GarV-D and GarV-X particles. Red and blue arrows indicate the 5′ end nucleotide of the PCR products of GarV-D and GarV-X, respectively. Number above the arrow indicates the number of ends detected. (PDF) [file ppat.1011457.s004.pdf]

# S5 Fig

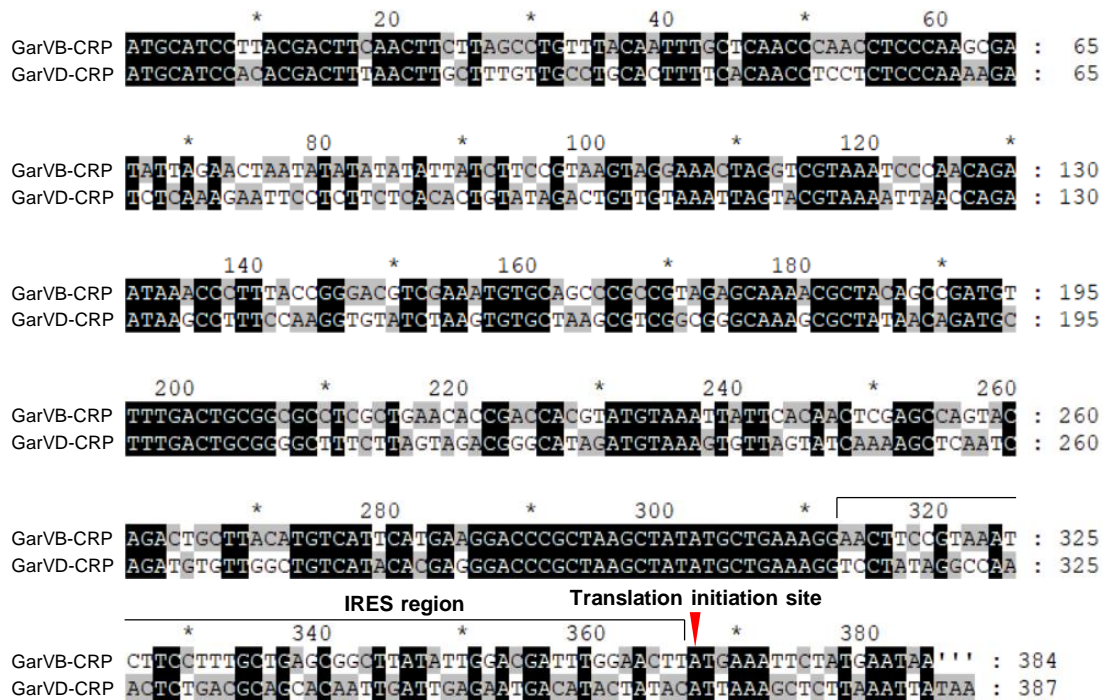

Supplement: S5 Fig — The positions of IRES and translation initiation site in GarV-B CRP are indicated. (PDF) [file ppat.1011457.s005.pdf]

# S6 Fig

A

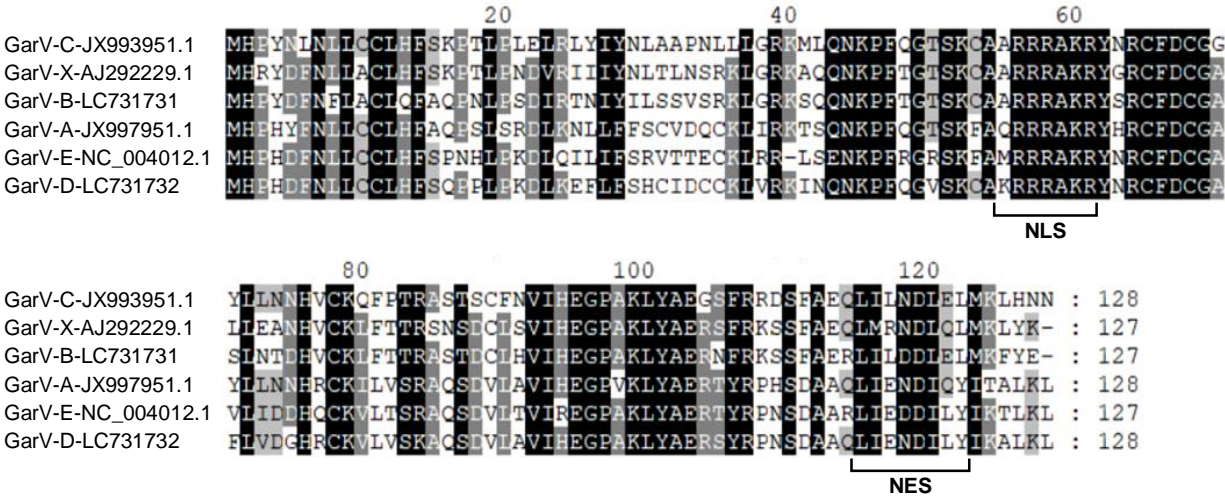

B

GarV-C CRP

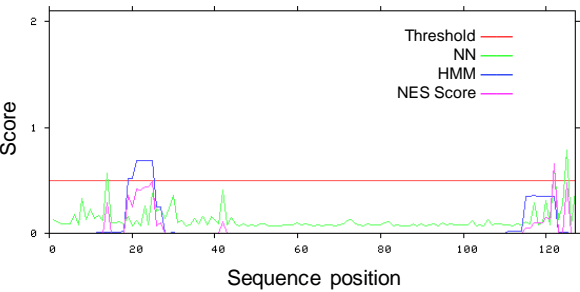

GarV-A CRP

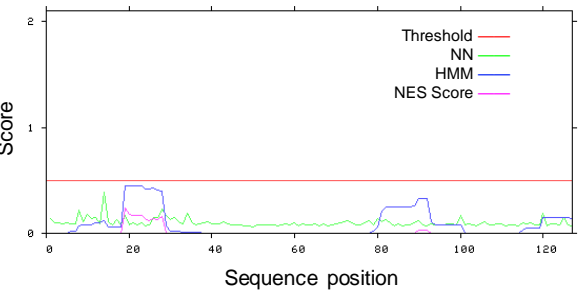

GarV-X CRP

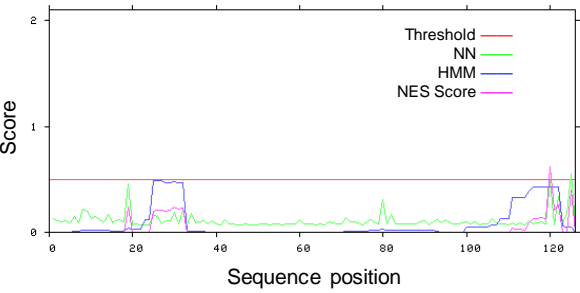

GarV-E CRP

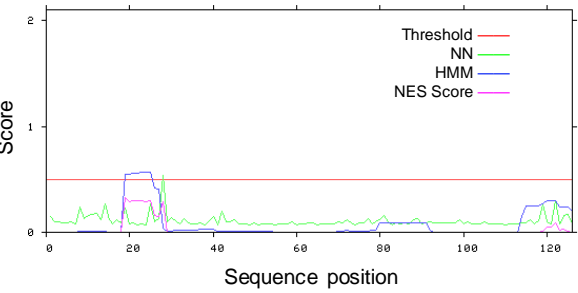

GarV-B CRP

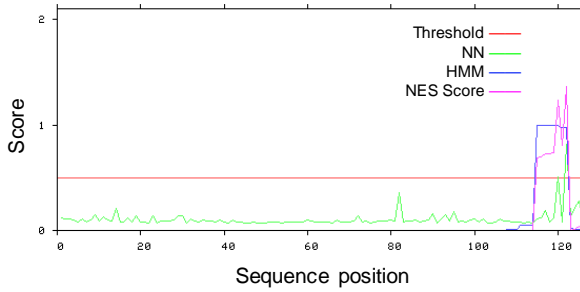

GarV-D CRP

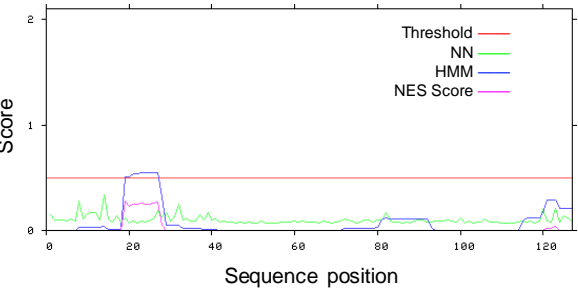

Supplement: S6 Fig — (A) Map of predicted nuclear localization signal (NLS) and nuclear export signal (NES) in the CRP amino acid sequences. (B) Prediction of NES motifs in CRPs of six allexiviruses by the program NetNES 1.1. Here, residues with NES scores higher than the threshold (red line) were predicted as putative NESs. (PDF) [file ppat.1011457.s006.pdf]

S7 Fig

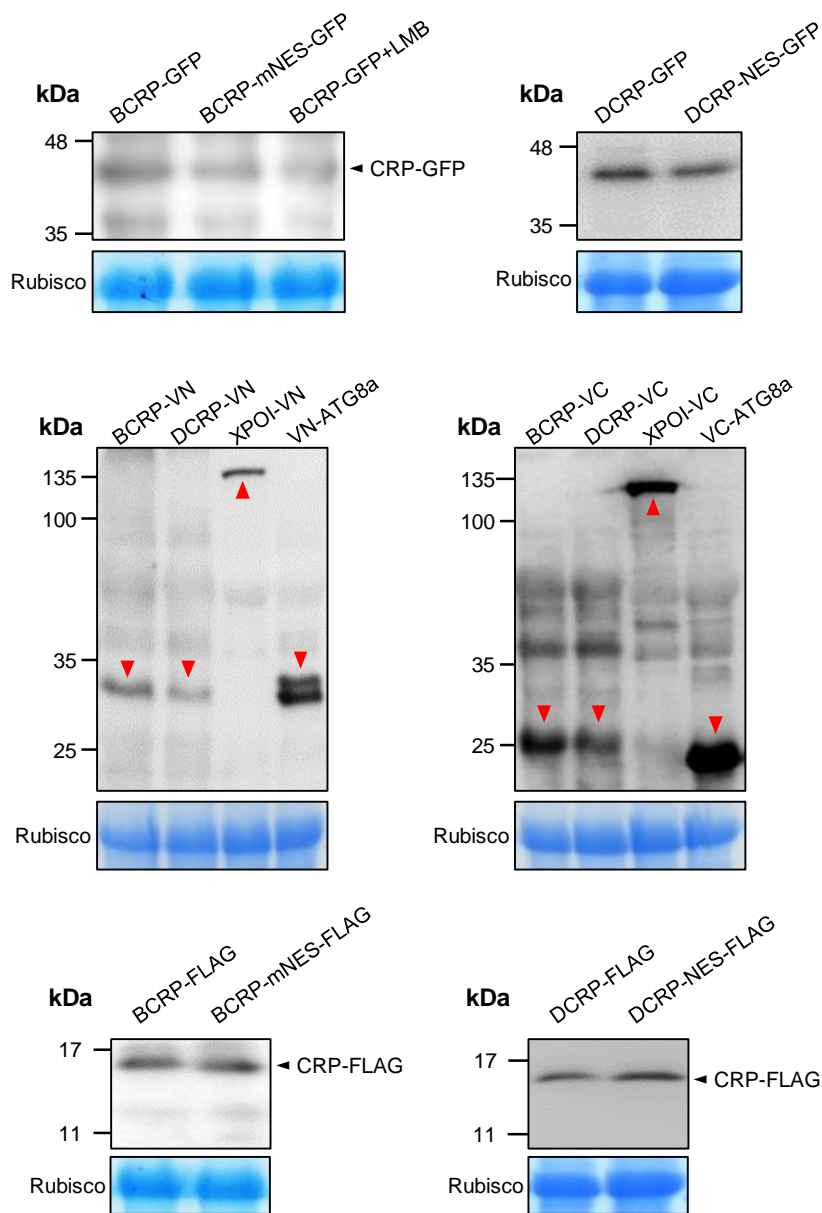

Supplement: S7 Fig — GFP- or mVenus (VN or VC)-fused proteins were detected using anti-GFP (anti-mVenus) polyclonal antibodies. The FLAG-tagged CRPs were detected using an anti-FLAG antibody. LMB, leptomycin B. (PDF) [file ppat.1011457.s007.pdf]

S8 Fig

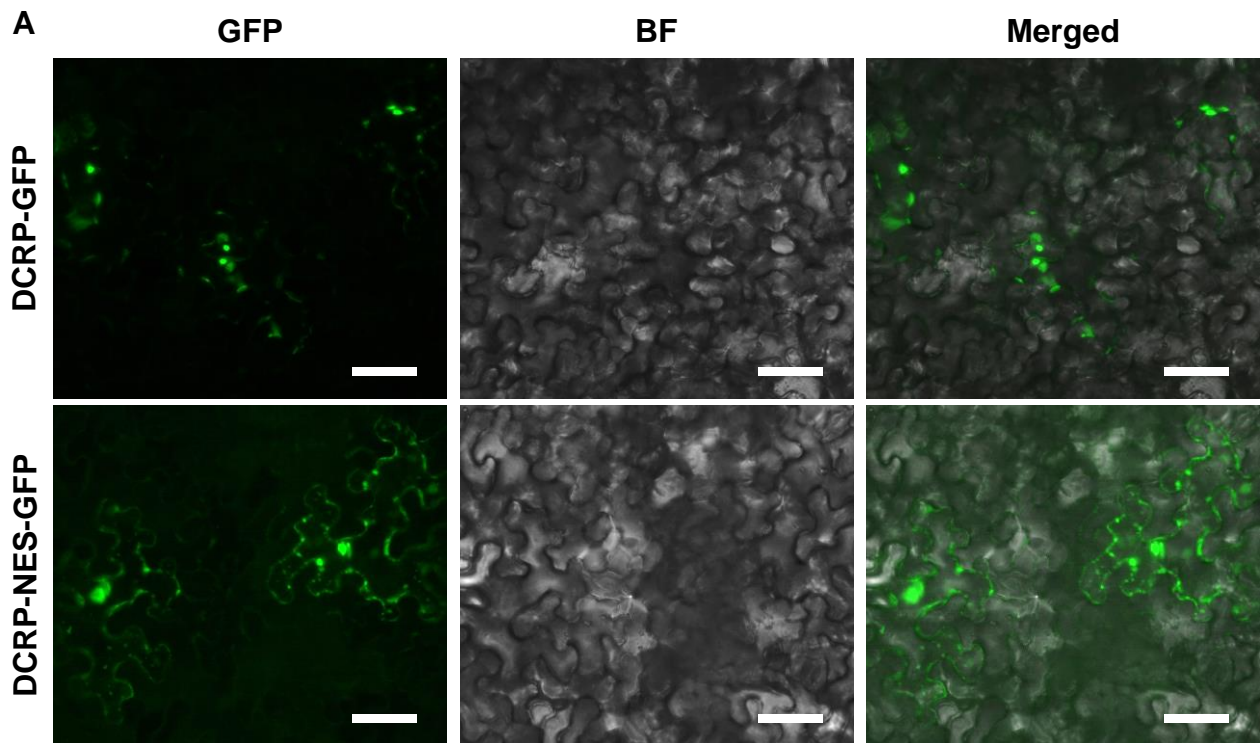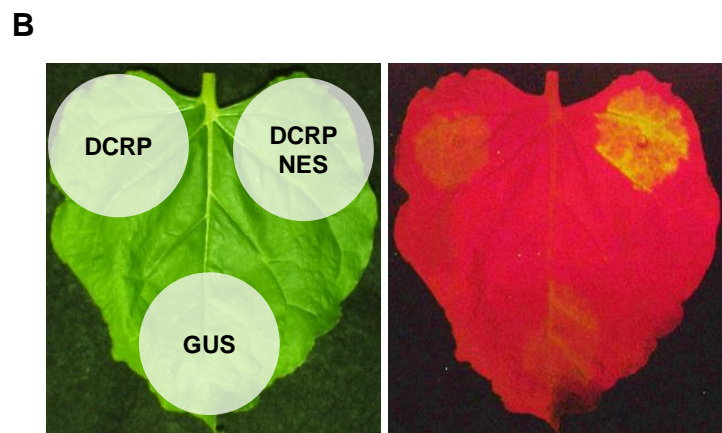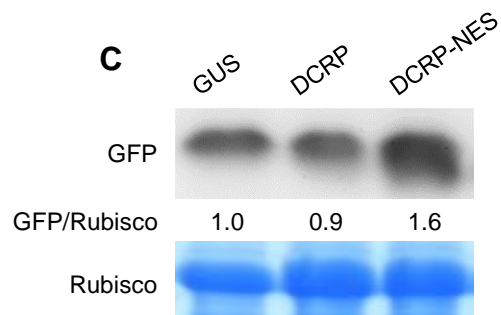

Supplement: S8 Fig — (A) Effect of the NES insertion in DCRP on the DCRP intracellular localization. The sequenceregion of DCRP corresponding to NES was replaced with that of BCRP (S6A Fig) to construct DCRP-NES. GFP-fused DCRP and DCRP-NES were expressed in N. benthamiana via agroinfiltration. Subcellular localization of the GFP fusion proteins were observed. Scale bars: 50 μm. (B) RSS activity of DCRP-NES. The RSS activities of GUS, FLAG-tagged DCRP and DCRP-NES were compared as described in the Fig 2 legend. (C) The GFP accumulation levels in the agroinfiltrated tissues were analyzed by western blot analysis. The expression of GFP-tagged or FLAG-tagged DCRP mutants were confirmed by western blot analysis as shown in S7 Fig. (PDF) [file ppat.1011457.s008.pdf]

# S9 Fig

DsRed-ATG8a + GUS

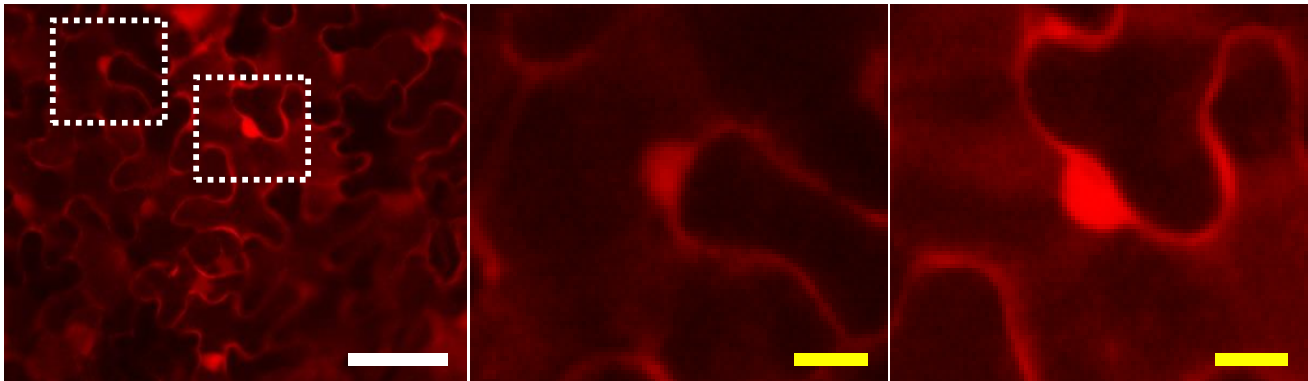

DsRed-ATG8a + BCP

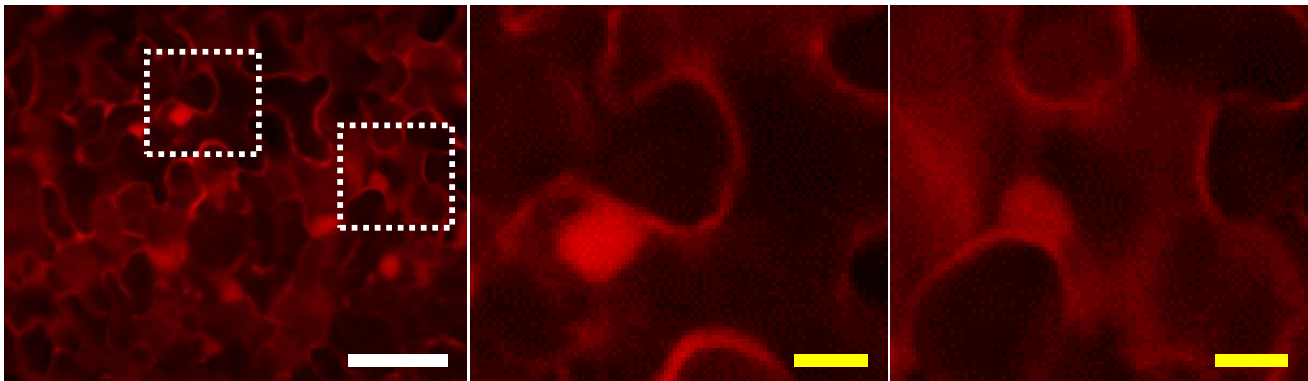

DsRed-ATG8a + DCP

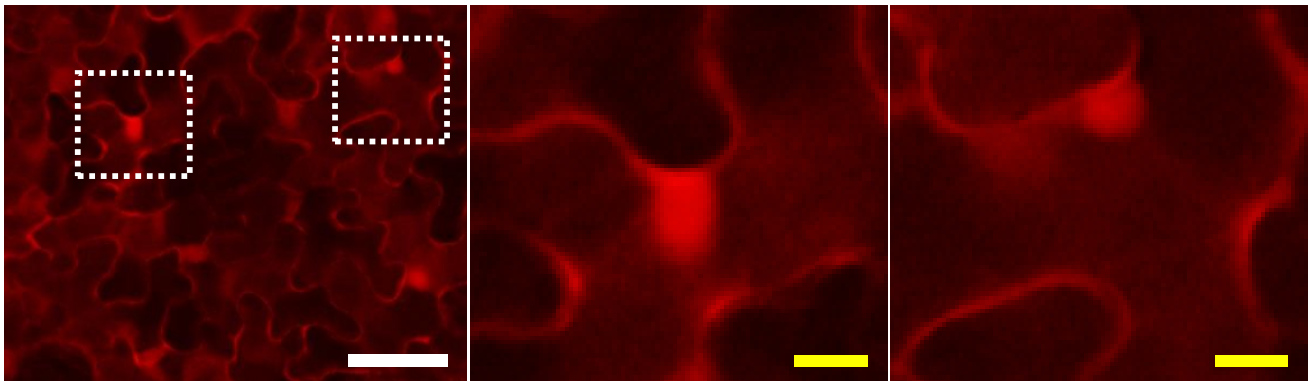

Supplement: S9 Fig — CP and DsRed-ATG8a were co-expressed in N. benthamiana leaves via agroinfiltration. DsRed fluorescence was observed as described in the Fig 5 legend. The two images on the right are close-ups of the white frame in the leftmost image. Scale bars: 50 μm (white), 10 μm (yellow). (PDF) [file ppat.1011457.s009.pdf]

S10 Fig

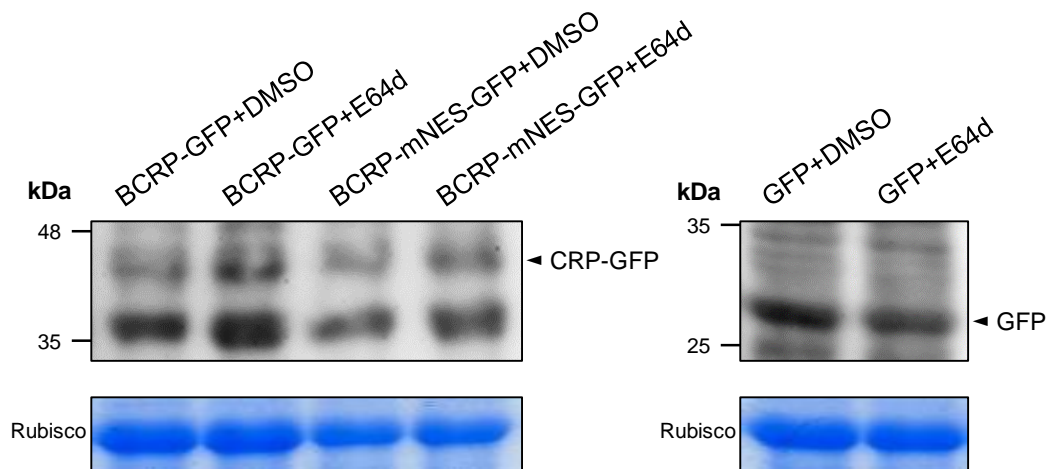

Supplement: S10 Fig — E64d (20 μM) was applied to the leaf tissues where CRPs had been expressed as described in Fig 6. The levels of the CRP-GFP accumulation were determined by western blot analysis at 16 h after the E64d treatment. (PDF) [file ppat.1011457.s010.pdf]

# S11 Fig

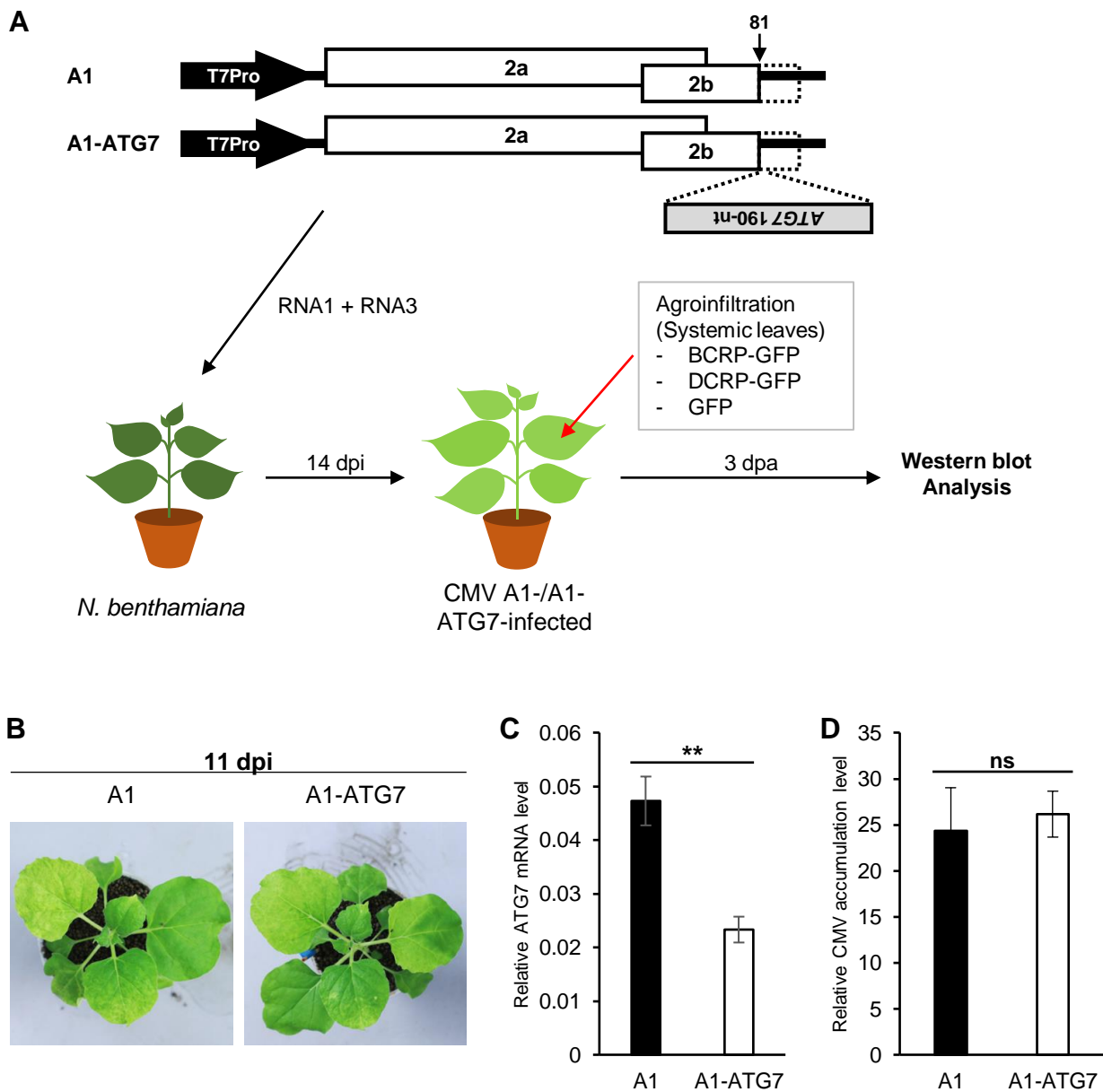

Supplement: S11 Fig — (A) Flowchart of ATG7 silencing in N. benthamiana. A partial ATG7 sequence (190-nt) was inserted into the A1 vector (A1-ATG7), and the in vitro transcripts of CMV RNA1, RNA3 and the recombinant A1 were used to inoculate to N. benthamiana plants. At 14 dpi, the CRP-GFP genes were expressed in systemically infected upper leaves by agroinfiltration, and the accumulation levels of CRP-GFP were estimated at 3 dpa by western blot analysis. (B) Symptoms of A1- and A1-ATG7-inoculated N. benthamiana plants. The pictures were taken at 11 dpi. (C, D) Comparison of the levels of ATG7 mRNA (C) and CMV RNA (D) between A1-infected and A1-ATG7-infected N. benthamiana plants. Real-time RT-PCRs were conducted at 14 dpi to measure the ATG7 mRNA and CMV RNA accumulation levels (n = 4). Two-sided Student’s t-test was performed for significant differences (**P < 0.01, ns: not significant). (PDF) [file ppat.1011457.s011.pdf]

S12 Fig

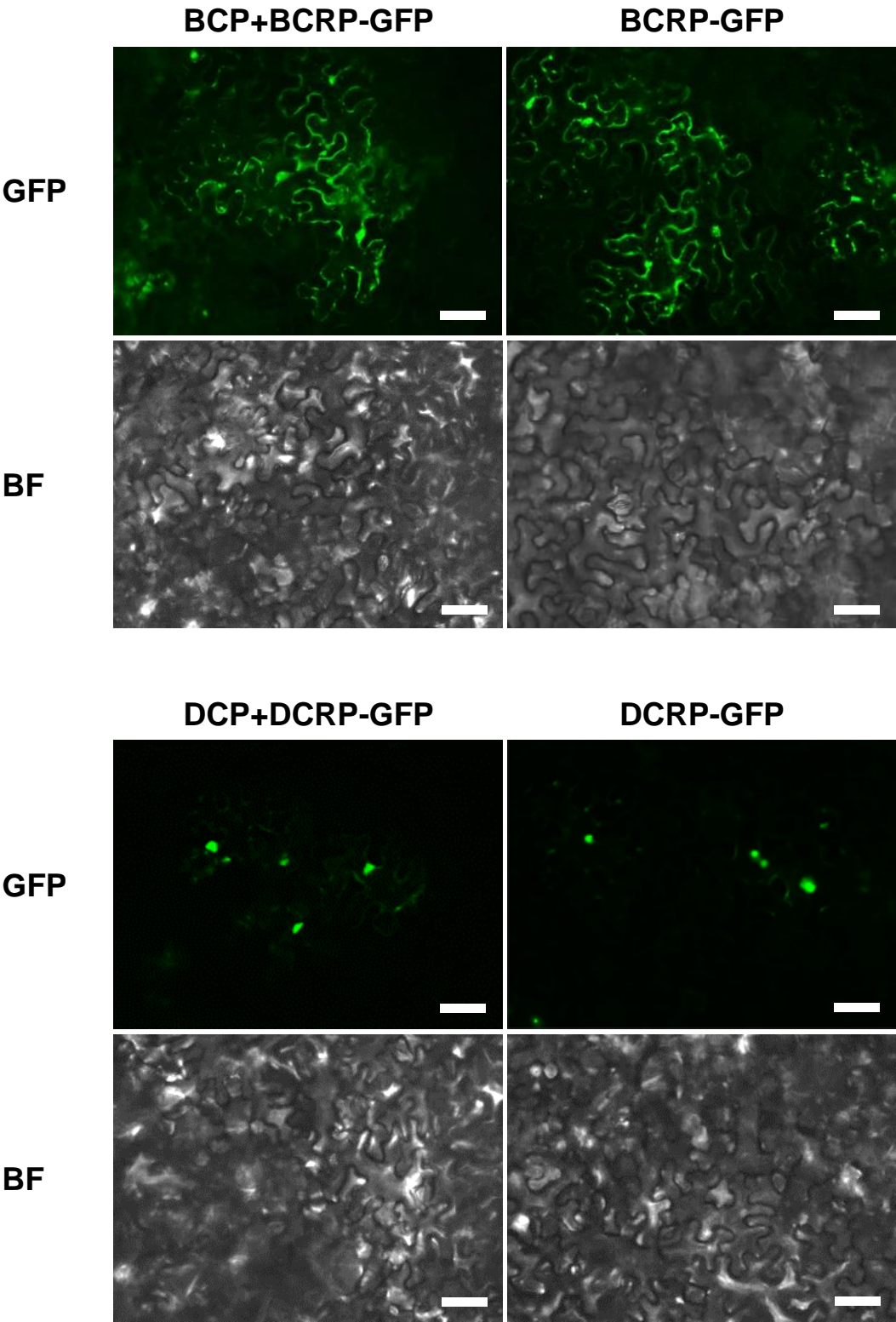

Supplement: S12 Fig — BCRP-GFP and DCRP-GFP were expressed with or without CP by agroinfiltration. The intracellular localization of CRP was observed using Leica DMI 6000B. Scale bars: 50 μm. (PDF) [file ppat.1011457.s012.pdf]

# S13 Fig

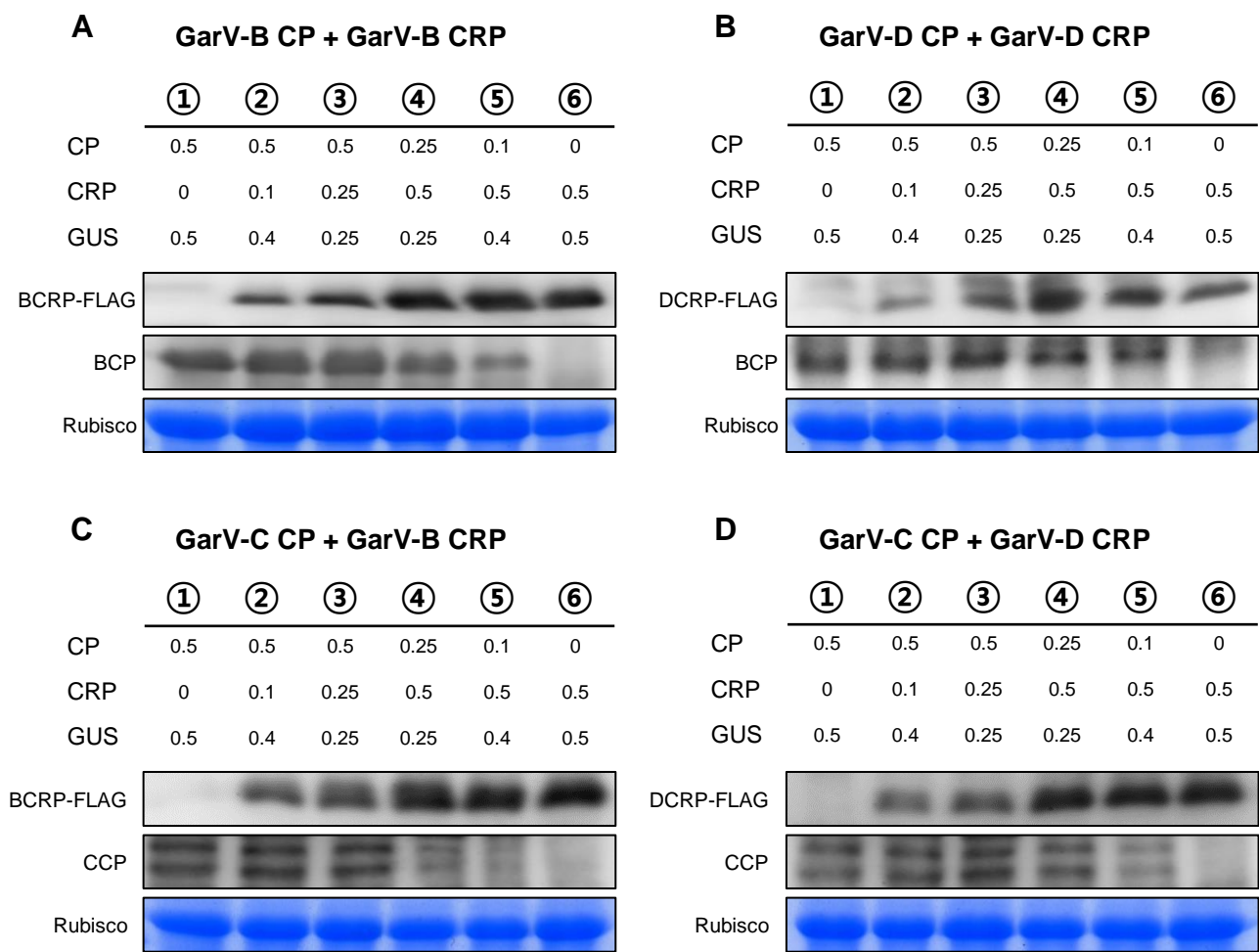

Supplement: S13 Fig — As indicated in the legends of Figs 7 and 8, CP and CRP-FLAG were co-expressed in N. benthamiana leaves by agroinfiltration using the bacterial suspension containing either [BCP+BCRP] (A), [DCP+DCRP] (B), [CCP+BCRP] (C) or [CCP+DCRP] (D). The amount of each construct in the infiltrated Agrobacterium suspension is shown at the top of each blot as OD600 values of the bacteria (1.0 for total). Western blot analyses were conducted to detect CP and CRP-FLAG using anti-CP antibodies and an anti-FLAG antibody, respectively. (PDF) [file ppat.1011457.s013.pdf]

S14 Fig

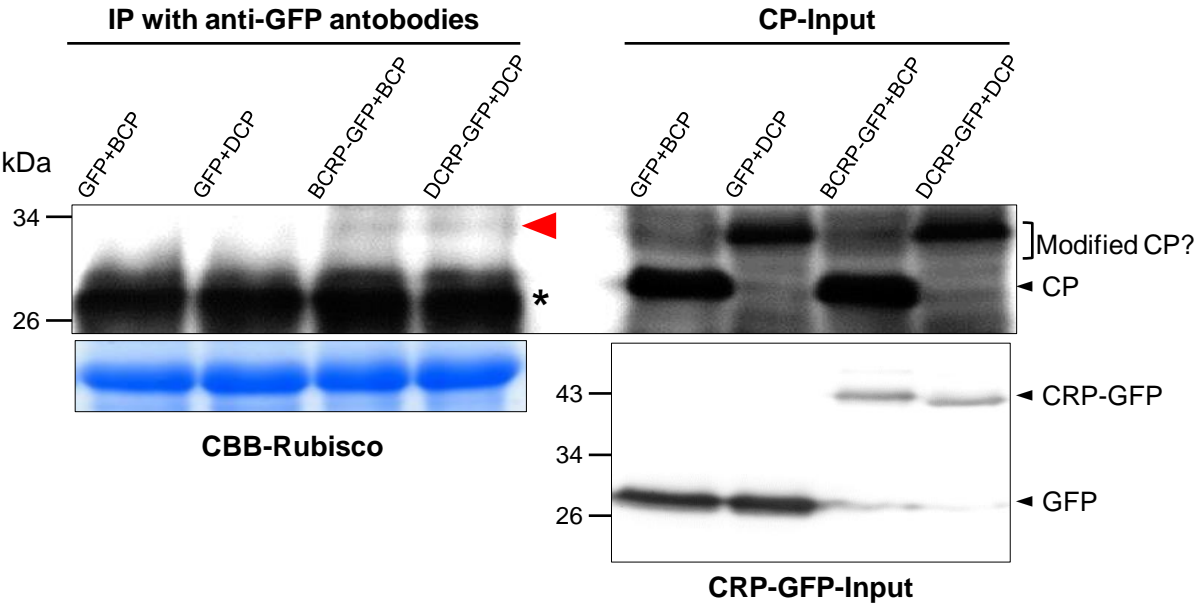

Supplement: S14 Fig — The CP/CRP-GFP complexes were first immunoprecipitated using anti-GFP antibodies, and then CP in the precipitant was detected by western blot analyses using anti-CP antibodies. The red arrowhead indicates the CP in the precipitant. The asterisk represents the light chain of anti-GFP antibodies. The CPs and CRP-GFPs in the total protein extracts, which were used for the IP experiments, were shown in the Input image. CBB-stained Rubisco large subunit is shown as a loading control. (PDF) [file ppat.1011457.s014.pdf]

## S15 Fig

**A**

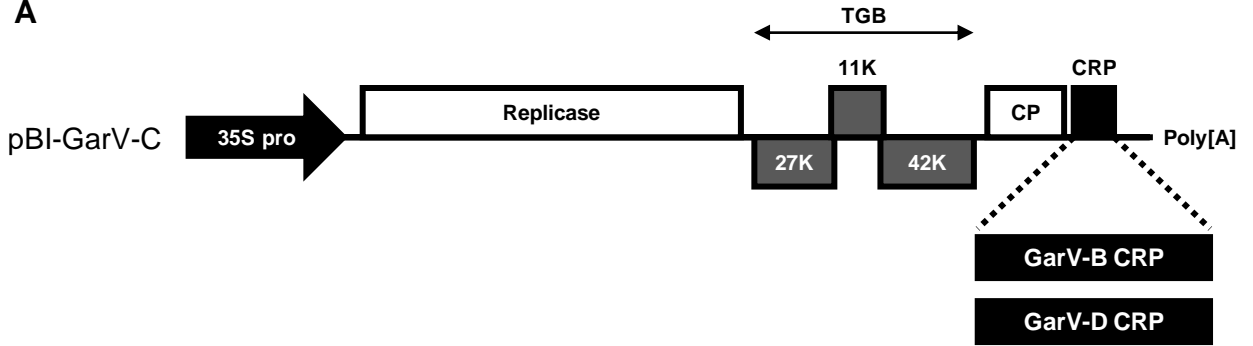

**B**

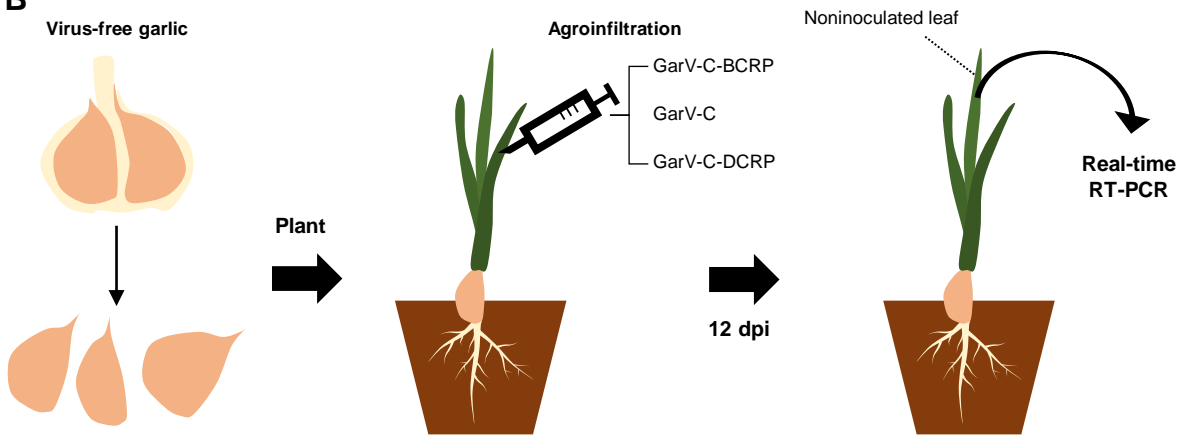

**C**

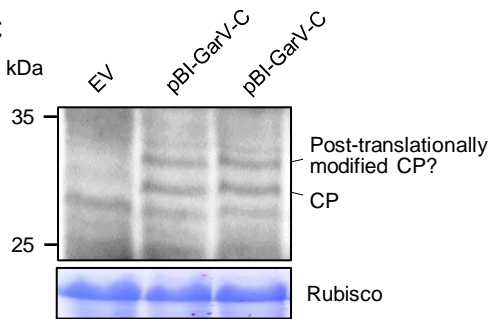

D

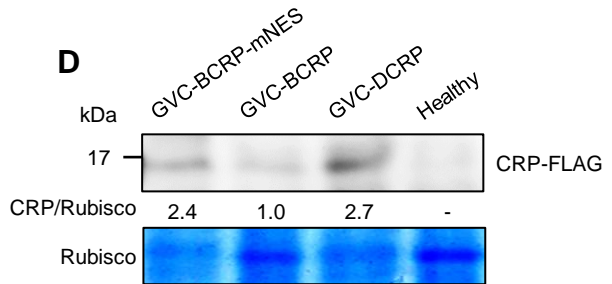

Supplement: S15 Fig — (A) Diagram of the GarV-C infectious clone containing either BCRP or DCRP. The binary vector pBI101 containing the 35S:GarV-C construct (pBI-GarV-C) was created, then the CRP of GarV-C was exchanged with either BCRP or DCRP. (B) Inoculation method of the GarV-C infectious clone. After shoots appeared from potted cloves of a virus-free garlic bulb, the leaves of each plant were infiltrated with Agrobacterium containing pBI-GarV-C using a 1-mL needleless syringe. Viral RNA in systemic leaves were quantified by real-time RT-PCR at 12 dpi. (C) Western blot for CP detection to confirm garlic infection by the recombinant GarV-Cs. The arrowhead indicates the CP size. The band just below the CP band seems to be also specific; it may be a processed CP. Leaves infiltrated with the pBI-GarV-C recombinant clones were harvested for western blot analysis at 7 dpa. The GarV-C CP was detected using anti-CP antibodies. Rubisco is shown as a loading control. (D) Verification of the CRP expression in the recombinant GarV-C-infected garlic plants at 7 dpa. The CRP region in pBI-GarV-C was replaced by either FLAG-tagged BCRP, BCRP-mNES or DCRP. The recombinant GarV-C constructs were then inoculated into garlic plants by agroinfiltration. The FLAG-tagged CRPs were detected by western blot analysis using an anti-FLAG antibody. Rubisco small subunit (Rubisco) is shown as a loading control. Note that the band for BCRP-mNES was stronger than that for BCRP, suggesting that the presence of a NES in CRP can affect CRP accumulation. (PDF) [file ppat.1011457.s015.pdf]

S16 Fig

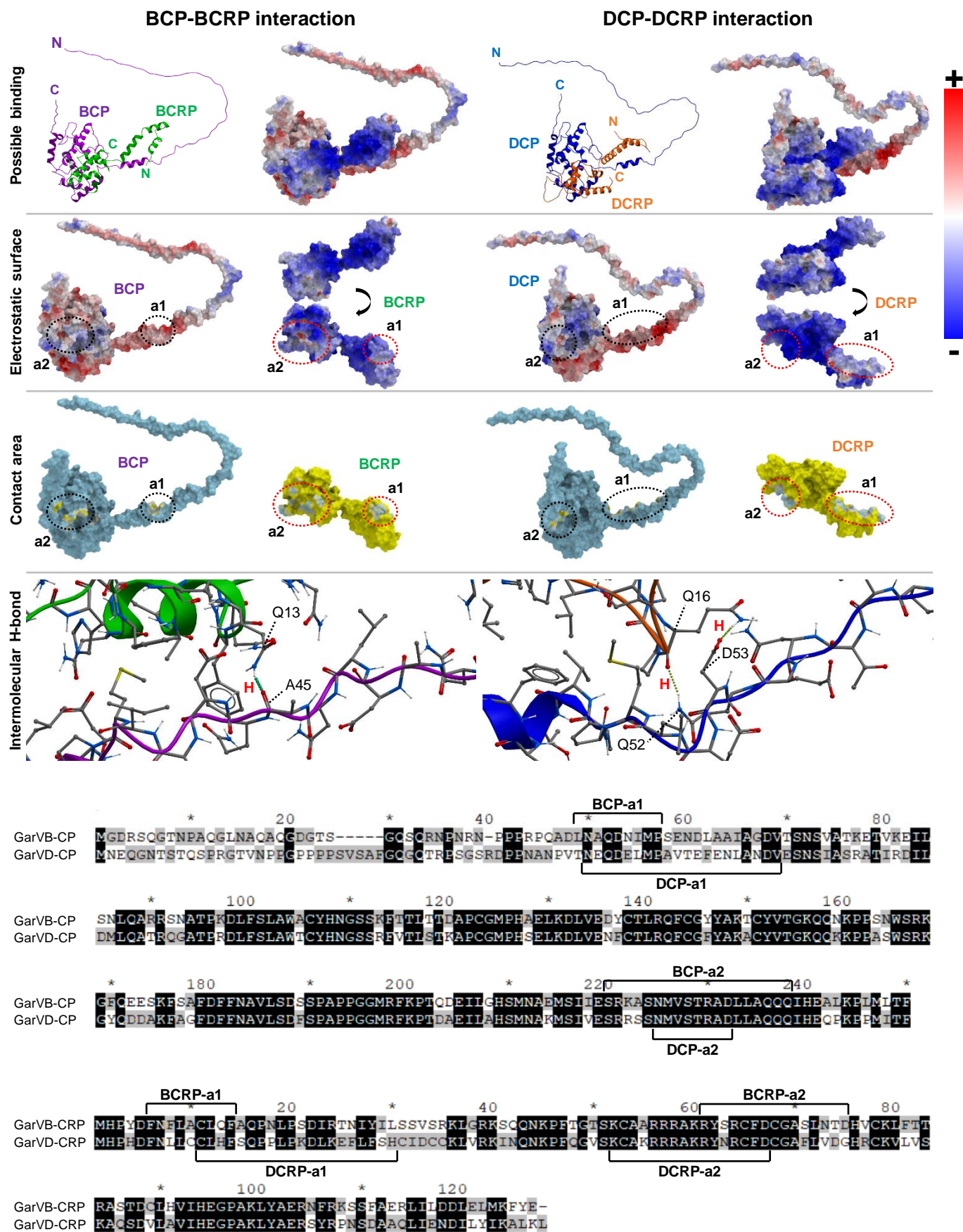

Supplement: S16 Fig — The predicted tertiary structures of BCP, DCP, BCRP and DCRP were constructed using AlphaFold v2.2.2 [49]. The N-terminal region of CP, which is variable among allexiviruses, was predicted not to form any secondary/tertiary structures regarded as an intrinsically disordered region. The in silico docking models for the CP-CRP association were constructed based on the Fast Fourier Transform (FFT) method in ICM-Pro v3.9 (Molsoft, San Diego, CA, USA), and the models with the lowest free energy were selected as the most stable associations. Note that the individual running of FFT docking for BCP-BCRP and DCP-DCRP resulted in very similar interaction models. The electrostatic surface and the contact areas were calculated and visualized in the ICM-Pro. The red- and black-dotted circles (a1 and a2) indicate the contact areas between CP and CRP. Close-up images of the a1 regions are shown to indicate the intermolecular hydrogen bonds (H). (PDF) [file ppat.1011457.s016.pdf]

S17 Fig

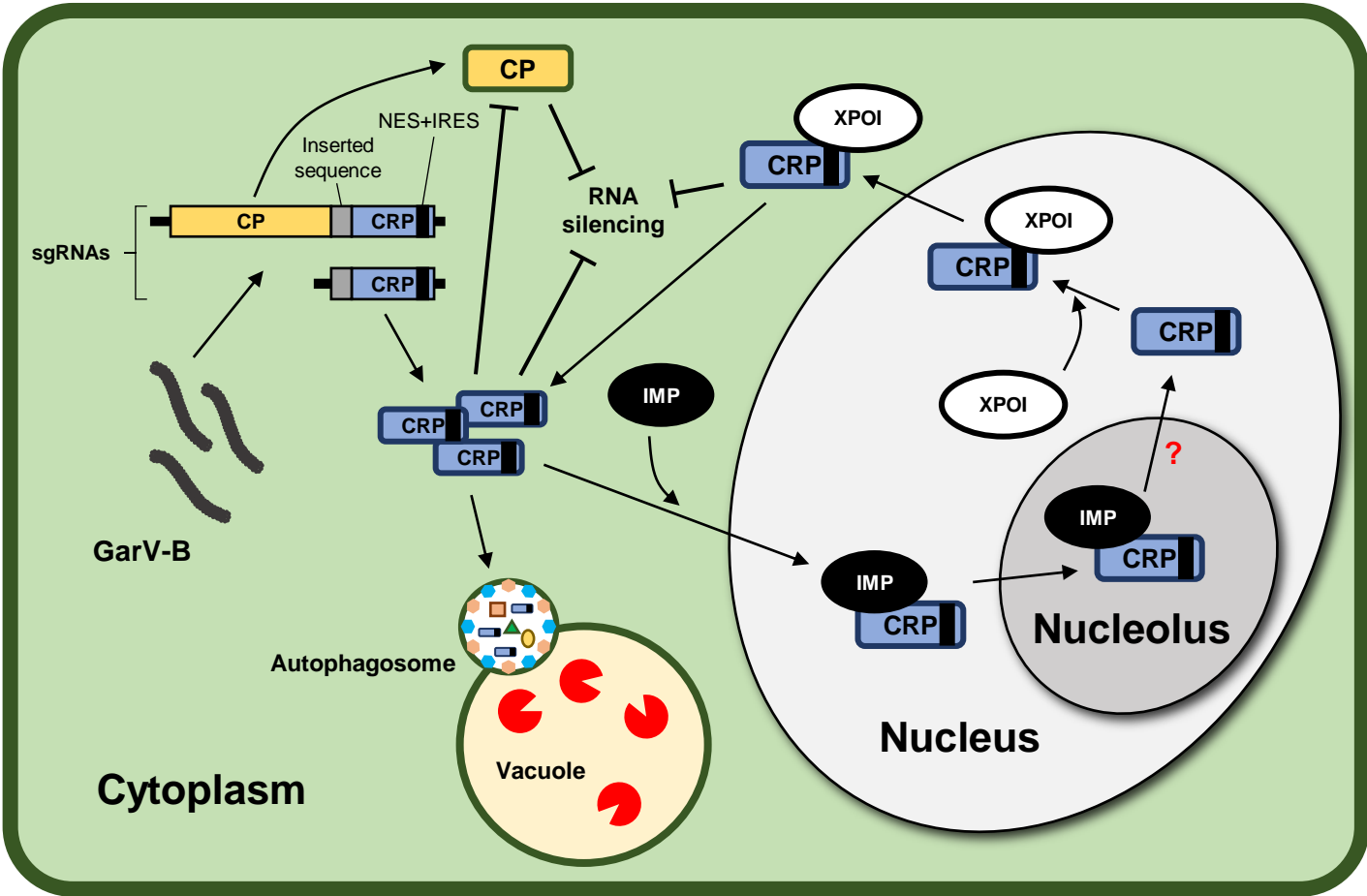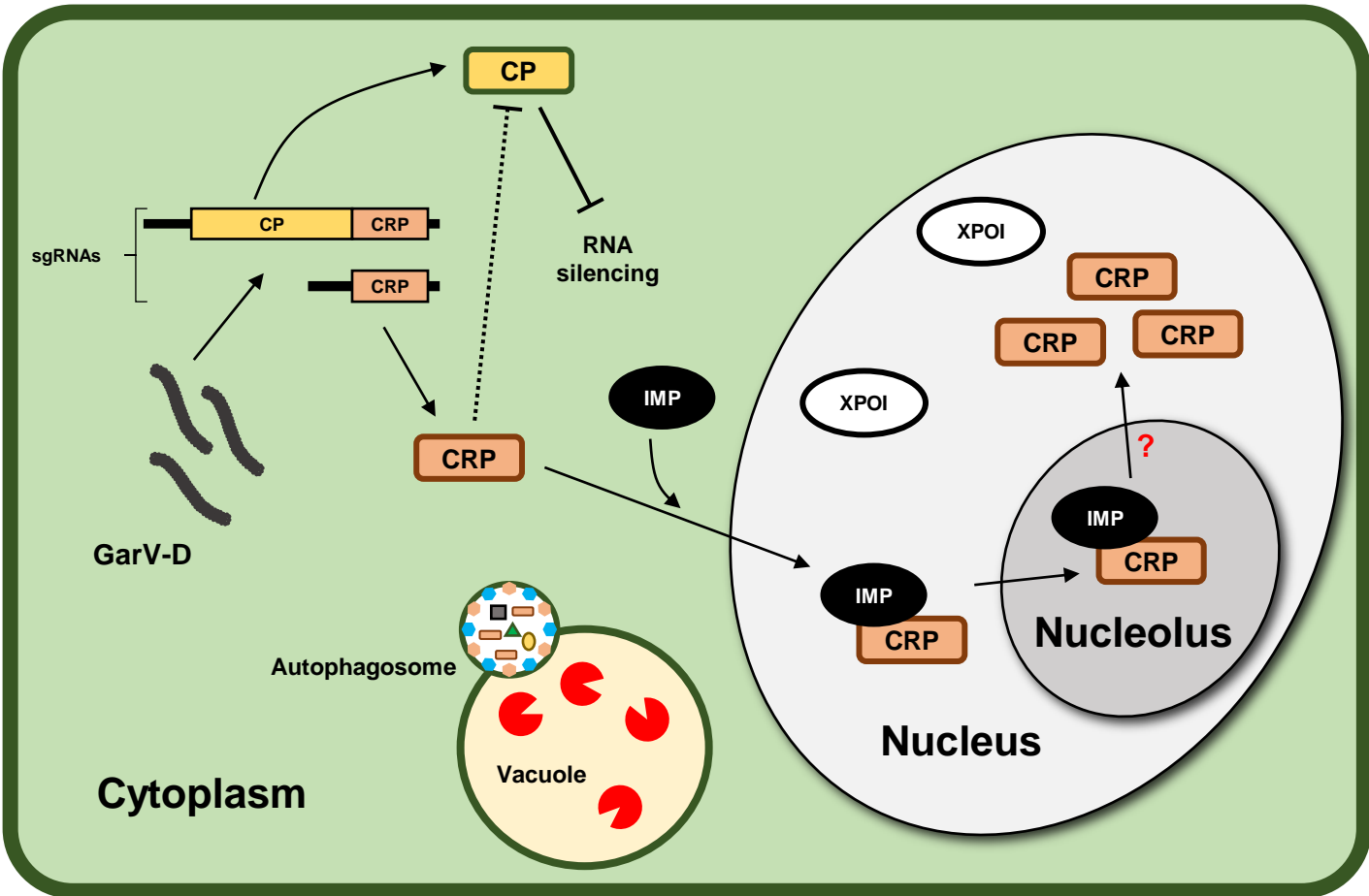

Supplement: S17 Fig — CP and CRP of GarV-B and GarV-D are expressed from sgRNAs. To avoid the effect of an IRES inside of the CRP ORF on CRP expression, GarV-B sgRNA-CRP may have acquired an IS between the CP and CRP ORFs to express CRP, while GarV-D sgRNA-CRP, which does not have an IRES, lacks IS. The CPs of the two allexiviruses can suppress RNA silencing in the cytoplasm. Although the CRPs can also function as RSSs in the cytoplasm, they are transported into the nucleus/nucleolus after translation in an importin (IMP)-dependent manner. GarV-B CRPs are then exported from the nucleus by interacting with XPOI; GarV-D CRPs, which lack an NES, are confined to the nucleus. The cytoplasmic GarV-B CRPs can function as an RSS, but because the CRPs and CPs interfere with each other, the two proteins may fail to suppress RNA silencing. Simultaneously, the cytoplasmic GarV-B CRPs are captured by autophagosome receptors and degraded via autophagy. On the other hand, GarV-D CRP may not able to efficiently suppress RNA silencing due to its low levels in the cytoplasm. However, in the nucleus, it escapes autophagy. In addition, because it is localized in the nucleus, the GarV-D CRP cannot interfere with the RSS activity of the CP. GarV-D CP can efficiently suppress RNA silencing. (PDF) [file ppat.1011457.s017.pdf]
