# Supplementary material for: Two mutually exclusive evolutionary scenarios for allexiviruses that overcome host RNA silencing and autophagy by regulating viral CRP expression
Source: PLoS Pathog. 2023 Jun 28;19(6):e1011457. doi: 10.1371/journal.ppat.1011457 (PMC10335701; doi:10.1371/journal.ppat.1011457)
Supplement: S2 Fig — GarV-B and GarV-D sequences were cloned into the plant expression vector pBE2113. (PDF) [file ppat.1011457.s002.pdf]

S2 Fig

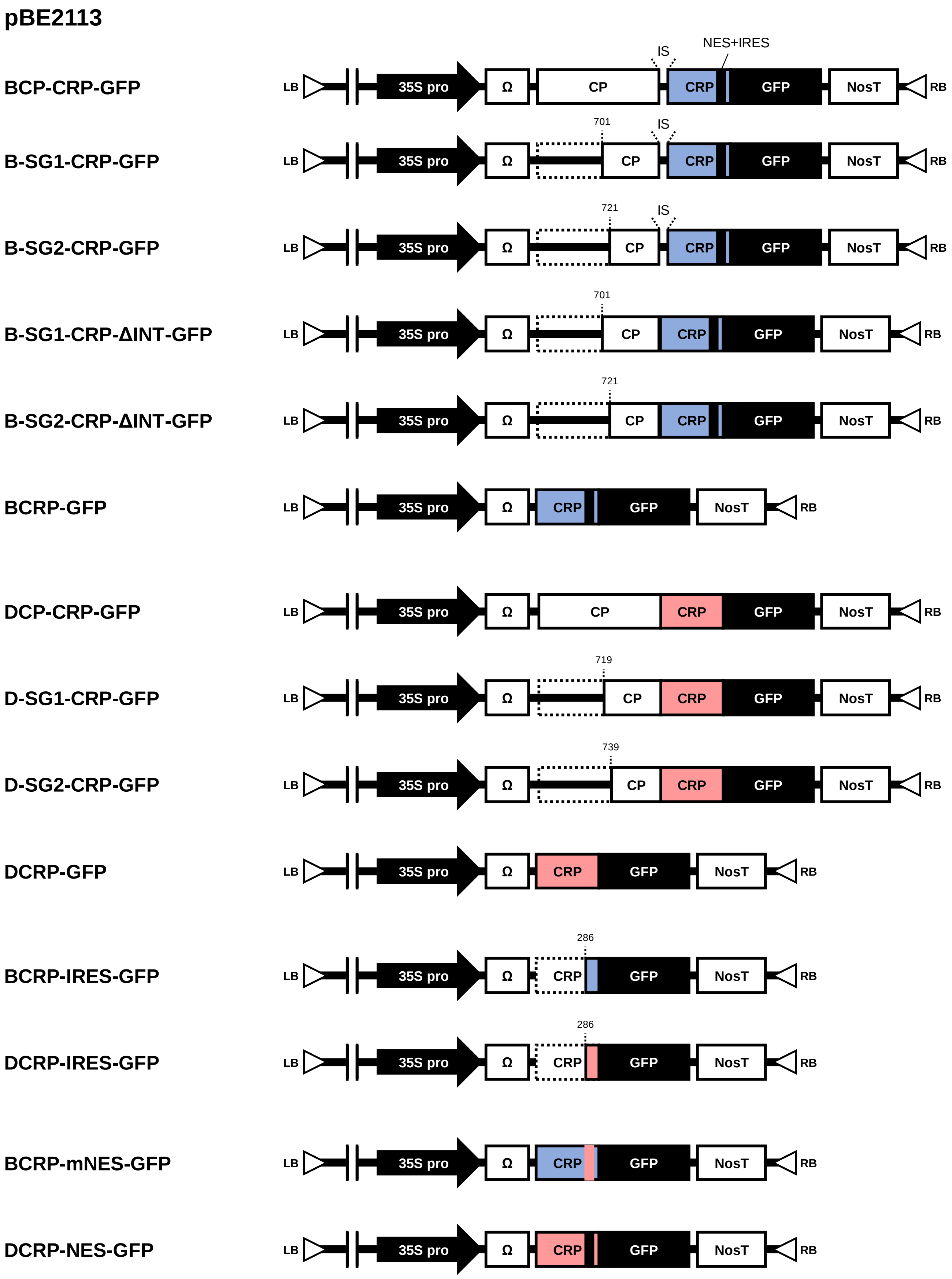
pBE2113
BCP-CRP-GFP
B-SG1-CRP-GFP
B-SG2-CRP-GFP
B-SG1-CRP-ΔINT-GFP
B-SG2-CRP-ΔINT-GFP
BCRP-GFP
DCP-CRP-GFP
D-SG1-CRP-GFP
D-SG2-CRP-GFP
DCRP-GFP
BCRP-IRES-GFP
DCRP-IRES-GFP
BCRP-mNES-GFP
DCRP-NES-GFP
LB
35S pro
Ω
CP
CRP
GFP
NosT
RB
IS
NES+IRES
701
721
719
739
286
