# Supplementary material for: Two mutually exclusive evolutionary scenarios for allexiviruses that overcome host RNA silencing and autophagy by regulating viral CRP expression
Source: PLoS Pathog. 2023 Jun 28;19(6):e1011457. doi: 10.1371/journal.ppat.1011457 (PMC10335701; doi:10.1371/journal.ppat.1011457)
Supplement: S1 Table — (PDF) [file ppat.1011457.s018.pdf]

S1 Table. Primers used in this study

F/R[a]	Primer name	Sequence (5´-3´)	Purpose
F	GVB-CP5-X	CGTCTAGAATGGGAGACAGGTCTCAAGG	Cloning of pBE2113-BCP
R	GVB-CP3-Sc	CGCGAGCTCCTAAAATGTAAGCATGAG	
F	GVD-CP5-X	CGTCTAGAATGAATGAACAAGGAAACAC	Cloning of pBE2113-DCP
R	GVD-CP3-Sc	CGCGAGCTCTCAGAATGTGATCATTGGAGG	
F	GVB-CRP5-Xb	CGCTCTAGAATGCATCCTTACGACTTCAAC	Cloning of pBE2113-BCRP-FLAG
R	GVB-Flg-CRP3-Sc	CGCGAGCTCTTACTTGTCATCGTCGTCCTTGTAGTCTTCATARAA	
R	GVB-CRP-S65T-3	AGTTCTTCTCCTTTACTCATTTCATAGAATTTCATAAGTTC	Cloning of pBE2113-BCRP-GFP
F	GVD-CRP5-Xb	CGTCTAGAATGCATCCACACGACTTTAA	Cloning of pBE2113-DCRP-FLAG
R	GVD-Flg-CRP3-Sc	CGCGAGCTCTTACTTGTCATCGTCGTCCTTGTAGTCTAATTTAAGA GCTTTAATGTAT	
R	GVD-CRP-S65T-3	AGTTCTTCTCCTTTACTCATTAATTTAAGAGCTTTAATGT	Cloning of pBE2113-DCRP-GFP
F	GB-CP5-Cla	CGGCATCGATATGGAGACAGGTCGCAAG	Cloning of PVX-BCP-CRP-FLAG
F	GB-CRP5-Cla	CCATCGATATGCATCCTTACGACTTCAACTTC	Cloning of PVX-BCRP-FLAG
R	GB-CRP3-Flg-Sal	CGCGTCGACTTACTTGTCATCGTCGTCC	
F	GD-CP5-Cla	CGGCATCGATATGAATGAACAAGGAAACAC	Cloning of PVX-DCP-CRP-FLAG
F	GD-CRP5-Cla	CGGCATCGATATGCATCCACACGACTTTAA	Cloning of PVX-DCRP-FLAG
R	GD-CRP3-Flg-Xho	CGGCCTCGAGTTACTTGTCATCGTCGTCC	
F	CRP-B-ATG1-5	GCGTCTAGAATGTCTATAATTGAGTCACG	Cloning of pBE2113-B-SG0-CRP-GFP
F	CRP-B-ATG2-5	GCGTCTAGAATGAAGCACTAAAACCACTC	Cloning of pBE2113-B-SG1-CRP-GFP
F	CRP-B-ATG3-5	GCGTCTAGAATGCTTACATTTTAGAAATCATTG	Cloning of pBE2113-B-SG2-CRP-GFP
F	CRP-D-ATG1-5	GCGTCTAGAATGTCAATTGTGGAATCCC	Cloning of pBE2113-D-SG0-CRP-GFP
F	CRP-D-ATG2-5	GCGTCTAGAATGAACAGCCTAAGCCTCCA	Cloning of pBE2113-D-SG1-CRP-GFP
F	CRP-D-ATG3-5	GCGTCTAGAATGATCACATTCTGATGCAT	Cloning of pBE2113-D-SG2-CRP-GFP
F	CRP-IRES-5-Xb	CGCTCTAGACTAAGCTATACGCTGAAAGG	Cloning of pBE2113 vector carring BCRP-IRES-GFP or DCRP-IRES-GFP
F	GVB-DELint-5	CCACTCATGCTTACATTTTAATGCATCCTTACGACTTCAA	Cloning of pBE2113 vector carring of B-SG1-CRP-ΔINT-GFP or B-SG2-CRP-ΔINT-GFP
R	GVB-DELint-3	TAAAATGTAAGCATGAGTGG	
F	GVB-dNES-5	TAAATCTTCCTTTGCTGAGCGATTGATTGAGAATGACATACTATAC ATGAAATTCTATGAAATGAGT	Cloning of pBE2113-BCRP-mNES-GFP
R	GVB-dNES-3	CGCTCAGCAAAGGAAGATTTA	
R	GVB-dNES-3-Sc	CGCGAGCTCTTATTCATAGAATTTCATGTA	Cloning of pBE2113-BCRP-mNES
F	S65T-5	ATGAGTAAAGGAGAAGAACT	Construction of GFP-fused BCRP and DCRP
R	S65T-3-Sc	CGCGAGCTCATGAAGTGACAGATAGTTATTTGTATAGTTCATCC	
F	GVB-CRP-5-Sm	TCCCCGGGATGCATCCTTACGACTTCAAC	Cloning of pBE2113-BCRP-VN and pBE2113-BCRP-VC
R	GVB-CRP-3-Nostp-Sp	ATACTAGTTTCATAGAATTTCATAAGTTC	
F	GVD-CRP-5-Sm	TCCCCGGGATGCATCCACACGACTTTAACT	Cloning of pBE2113-DCRP-VN and pBE2113-DCRP-VC
R	GVD-CRP-3-Nostp-Sp	ATACTAGTTAATTTAAGAGCTTTAATGTATAG	
R	BCRP-mNES-FLAG-Sp	GCACTAGTTTACTTGTCATCGTCGTCCTTGTAGTCTTCATAGAATT TCATGTA	Construction of BCRP-mNES-FLAG
R	FLAG-3-Sc	CGCGAGCTCTTACTTGTCATCGTCGTCC	Cloning of pBE2113-BCRP-mNES-FLAG
F	DCRP-NES-F	ATATTGGACGATTTGGAACTTATTAAAGCTCTTAAATTA	Cloning of pBE2113-DCRP-NES-FLAG and pBE2113-DCRP-NES-GFP
R	DCRP-NES-R	AAGTTCCAAATCGTCCAATATCAATTGTGCTGCGTCAGAG	
F	NbXPO1-5-Sm	TCCCCGGGATGGCGGCGGATAAGCTTAGAGATT	Cloning of pBE2113-XPOI-VN and pBE2113-XPOI-VC
R	NbXPO1-NoStp-3-Sp	ATACTAGTTGAGTCAACCATTTCATCCTGTAT	
F	DsRed-5-Xb	GCTCTAGAATGGACAACACCGAGGACGT	Cloning of pBE2113-DsRed-ATG8a
R	DsRed-ATG8a-3	AATTTGAAGGAGCTTTTGGCCATCTGGGAGCCGGAGTGGCG	
F	ATG8a-5	ATGGCCAAAAGCTCCTTCAAATT	
R	ATG8a-3-Sc	CGCGAGCTCTTAGAACGATCCGAATGTATTCTCTC	
F	mVenus-N-5-Sm	GCCCCGGGATGGTGAGCAAGGGCGAGGAG	Cloning of pBE2113-VN-ATG8a and pBE2113-VC-ATG8a
F	mVenus-C-5-Xb	GCTCTAGAATGACCGCCGACAAGCAGAAG	
R	VN-ATG8a-3	AATTTGAAGGAGCTTTGGCCATGTTAACCCCGGTTCTAGAG	
R	VC-ATG8a-3	AATTTGAAGGAGCTTTTGGCCATCTTGTACAGCTCGTCCATGC	
F	ATG7-5-190-Ml	CGCACGCGTTTGCTCCCATCCTCAAGTATC	Cloning of A1-ATG7
R	ATG7-3-190-St	CGAGGCCTCCTTAAGGAGGCTCTGCTTATC	
F	GVC-CP-5-Xb	CGTCTAGAATGAGTGGAGACAGCCTATC	Cloning of pBE2113-CCP
R	GVC-CP-3-Sc	CGCGAGCTCTCAAAACGTTAACATGAGAGGC	
F	Alle-CP5-750	TGGRCNTGCTACCACAAYGG	Real-time RT-PCR for GarV-C CP
R	Alle-CP3-750	CCYTTCAGCATATAGCTTAGC	Real-time RT-PCR for GarV-C CP, cDNA synthesis for 5' RACE
F	S65T-5-168	TCACGGCAGACAAACAAAAG	Real-time RT-PCR for GFP
R	S65T-3-168	AAAGGGCAGATTGTGTGGAC	
R	GVB-CRP3-80	GATAATATATATATATTAGTT	5´ RACE for detection of GarV-B sgRNA-CRP
R	GVD-CRP3-80	CAGTGTGAGAAGAGGAATTCT	5´ RACE for detection of GarV-D sgRNA-CRP
R	GVD-CRP3-60	GATCTTTTGGGAGAGGAGGTTG	
R	GVX-CRP3-60	CGTCGTTTGGAAGTGTTGGCTT	5´ RACE for detection of GarV-X sgRNA-CRP
F	Garlic-act-rt-F2	GAATTGTGAGCAACTGGGATGAC	Real-time RT-PCR for reference of garlic *(A. sativum)*
R	Garlic-act-rt-R2	GGAAAGCACAGCCTGGATAGC	
F	PVX-CP5-110	TTGACTTCTTTGATGGAGTCACC	Real-time RT-PCR for PVX CP
R	PVX-CP3-110	TTCAGACGGAGGTCTCATGAGC	
F	ATG7-5-108	TGGGGTGTTACTTCTGCAATGA	Real-time RT-PCR for *ATG7*
R	ATG7-3-108	ATGCAATAGGAGCAAGTCCCG	
F	CMV-DET-5-340	GTTGACGTCGAGCACCAACGC	Real-time RT-PCR for CMV
R	CMV-DET-3-340	TGGTCTCCTTTTGGAGGCCC	
F	Nb-L23-5-110	AAGGATGCCGTGAAGAAGATGT	Real-time RT-PCR for reference of *N. benthamiana*
R	Nb-L23-3-110	GCATCGTAGTCAGGAGTCAACC	

[a] F, Forword primers; R, Reverse primers
